# Formulas and algorithms for the length of a Farey sequence

**DOI:** 10.1038/s41598-021-99545-w

**Published:** 2021-11-15

**Authors:** Vladimir Sukhoy, Alexander Stoytchev

**Affiliations:** Department of Electrical and Computer Engineering, Iowa State University, Ames, IA 50011 USA

**Keywords:** Mathematics and computing, Computer science

## Abstract

This paper proves several novel formulas for the length of a Farey sequence of order *n*. The formulas use different trade-offs between iteration and recurrence and they range from simple to more complex. The paper also describes several iterative algorithms for computing the length of a Farey sequence based on these formulas. The algorithms are presented from the slowest to the fastest in order to explain the improvements in computational techniques from one version to another. The last algorithm in this progression runs in $O(n^{2/3})$ time and uses only $O(\sqrt{n})$ memory, which makes it the most efficient algorithm for computing $|F_n|$ described to date. With this algorithm we were able to compute the length of the Farey sequence of order $10^{18}$.

## Introduction

Farey sequences^1,2^ are related to the theory of prime numbers and they show up in many different scientific disciplines. Their fundamental properties, e.g., the mediant property, can be described with basic algebra. At the same time, Farey sequences are linked to unsolved mathematical mysteries, e.g., the Riemann hypothesis^3^. These sequences are also tied to the Stern–Brocot tree, which could be used to find the best rational approximations for irrational numbers^4^. Recently, the elements of a Farey sequence have been linked to the singularities^5^ of the Inverse Chirp Z-Transform (ICZT), which is a generalization^6^ of the Inverse Fast Fourier Transform (IFFT).

A Farey sequence of order *n*, which is denoted by $F_n$, is a sequence formed by all irreducible fractions $\frac {p}{q}$ between 0 and 1 for which the denominator *q* is between 1 and *n*, i.e., $F_n = \big \{ \frac {p}{q} \text { s.t. } q \in \{1, 2, \dotsc , n\},\, p \in \{0, 1, 2, \dotsc , q\}, \text { and } \text {gcd}(p,q)=1 \big \}$. By convention, it is assumed that the elements of the sequence $F_n$ are sorted in increasing order. For example, the first five Farey sequences are:

$$\begin{aligned} F_1&= \left( \frac{0}{1}, \, \frac{1}{1} \right) , \\ F_2&= \left( \frac{0}{1}, \, \frac{1}{2}, \, \frac{1}{1} \right) , \\ F_3&= \left( \frac{0}{1}, \, \frac{1}{3}, \, \frac{1}{2}, \, \frac{2}{3}, \, \frac{1}{1} \right) , \\ F_4&= \left( \frac{0}{1}, \, \frac{1}{4}, \, \frac{1}{3}, \, \frac{1}{2}, \, \frac{2}{3}, \, \frac{3}{4}, \, \frac{1}{1} \right) , \\ F_5&= \left( \frac{0}{1}, \, \frac{1}{5}, \, \frac{1}{4}, \, \frac{1}{3}, \, \frac{2}{5}, \, \frac{1}{2}, \, \frac{3}{5}, \, \frac{2}{3}, \, \frac{3}{4}, \, \frac{4}{5}, \, \frac{1}{1} \right) . \end{aligned}$$

If $\frac {a}{b}$, $\frac {c}{d}$, and $\frac {p}{q}$ are any three adjacent elements of a Farey sequence, then the middle fraction is equal to the *mediant*^2,7^ of its neighbors $\frac {a}{b}$ and $\frac {p}{q}$, i.e.,

$$\begin{aligned} \frac{c}{d} = \frac{a + p}{b + q}. \end{aligned}$$

This property has been known to mathematicians for centuries^8,9^, but it received a name only after Farey stated it formally in a paper^1^ that he published in 1816. Previously, Haros^2,9^ had used the mediant property in 1802 to generate the tables of irreducible fractions between 0 and 1 for which the denominator was less than 100 (see Ref.^2^, p. 36). Cauchy published a formal proof of the mediant property^10^ for all *n* in 1816.

As the order *n* of the Farey sequence $F_n$ increases, the length of $F_n$ grows as a quadratic function of *n*. More specifically, $|F_n| \sim 3 n^{2} / \pi ^{2}$, where $|F_n|$ denotes the length of $F_n$ [2, p. 268] [7, p. 156] [4, p. 139]. However, no formula for computing the exact value of $|F_n|$ in *O*(1) time is known.

The length of $F_n$ can be computed by enumerating its elements. Algorithm S1 in Supplementary Section S1 gives the pseudo-code for an algorithm^11^ that uses the mediant property to enumerate all elements of a given Farey sequence. The algorithm also counts the elements and returns the sequence length. The computational complexity of this approach is $O(n^2)$, which makes it too slow and impractical for computing the value of $|F_n|$ for large *n*. The fast algorithms described in this paper do not use enumeration. Their computational complexities are summarized in Supplementary Sect. S2.

Increasing the value of *n* only adds new elements to the Farey sequence without removing any of them. That is, $F_{n-1}$ is a subsequence of $F_n$. To see this, consider the elements of the first five Farey sequences shown below, which are arranged with extra spacing between them. In this view the identical fractions are stacked vertically. The new elements that are added when the order is increased by 1 are highlighted in red. The length of each Farey sequence is shown at the end of each row. 
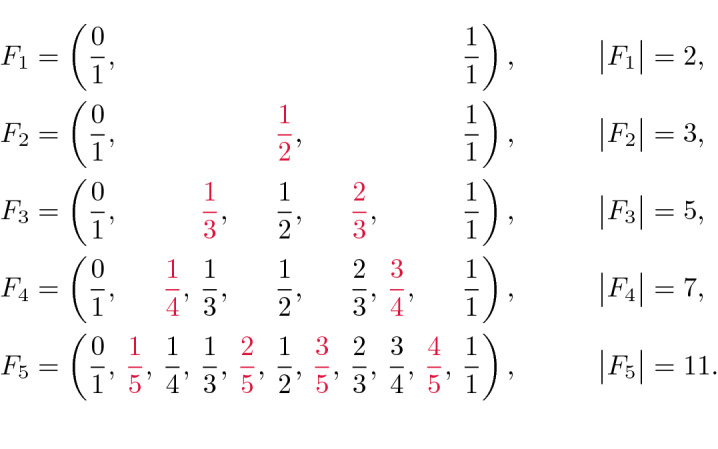


Each element of the set $F_n \setminus F_{n-1}$ is a fraction $\frac {k}{n}$ that is irreducible and lies between 0 and 1. Thus, the difference between $|F_n|$ and $|F_{n-1}|$ is equal to the number of integers between 1 and *n* that are coprime with *n*. By definition, this number is equal to the value of Euler’s totient function $\varphi (n)$. Therefore, an algorithm^12^ for computing the length of $F_n$ can also be used to calculate the sums of the values of Euler’s totient function for all integers between 1 and *n*.

**Figure 1:**
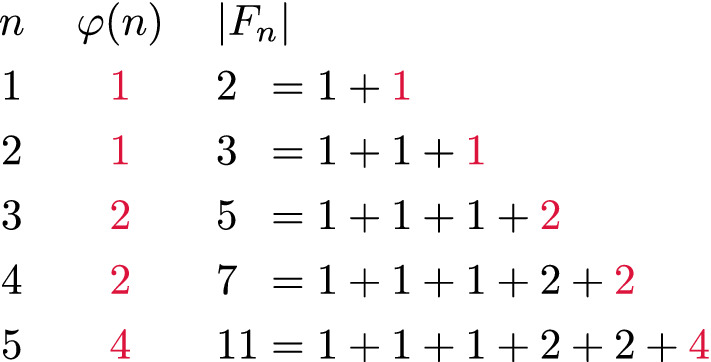
Visualization of the link between Euler’s totient function $\varphi (n)$ and the length of the Farey sequence $F_n$.

Figure 1 lists the values of Euler’s totients $\varphi (1)$, $\varphi (2)$, $\varphi (3)$, $\varphi (4)$, $\varphi (5)$ and expresses the values of $|F_1|$, $|F_2|$, $|F_3|$, $|F_4|$, and $|F_5|$ in terms of these totients. This example also illustrates the following well-known formula for computing $|F_n|$ that is proven in Supplementary Sect. S3:

1 $$\begin{aligned} |F_n|&= 1 + \sum _{k = 1}^n \varphi (k). \end{aligned}$$

This equation implies that the value of $\varphi (n)$ can be added to $| F_{n-1} |$ to obtain $|F_n|$, i.e., $|F_n| = |F_{n+1}| + \varphi (n)$.

The value of $|F_n|$ can also be expressed using the following recursive formula^13^:

2 $$\begin{aligned} |F_n| = \frac{(n + 3) n}{2} \, - \, \sum _{k=2}^n \left| F_{\lfloor n /k \rfloor }\right| . \end{aligned}$$

Supplementary Sections S4 and S5 prove this formula using basic algebra, mathematical induction, and a property of Euler’s totient function that was proven by Gauss^14^. Algorithm S11 in Supplementary Sect. S9 implements formula (2) using recursion and optimizes the repeated recursive calls using *memoization*^15^. That algorithm runs in $O(n \log n)$ time, which is still too slow for large values of *n*.

The algorithms described in this paper build upon these two formulas, extend them, and combine them in different ways. The next section summarizes the key mathematical insights that were used to derive the formulas. The rest of the paper describes the algorithms, proves their properties, and evaluates their run-time performance.

## Overview and formulas

This paper describes five different algorithms for computing the length of the Farey sequence $F_n$, where *n* is a parameter that specifies the order. The algorithms will be denoted with the letters A, B, C, D, and E. These letters will also be used as suffixes to form the names of the algorithms, e.g., FareyLengthA or FareyLengthB. This section briefly summarizes the formulas for the length of $F_n$ on which the algorithms are based.

Algorithm A is based on the following well-known formula:

3 $$\begin{aligned} |F_n| = 1 + \sum _{k=1}^n \varphi (k), \end{aligned}$$

which was illustrated with an example in the introduction. The algorithm uses a modified linear sieve that returns a list of prime numbers and an array with the smallest prime factor for each integer. From these values, another function computes Euler’s totients, which are then summed up to compute the length of $F_n$. This algorithm runs in *O*(*n*) time and uses *O*(*n*) memory.

The formula for algorithm B is derived from (3) by splitting the sum into three separate sums:

4 $$\begin{aligned} |F_n| = 1 + \sum _{k = 1}^{\lfloor \sqrt{n} \rfloor } \varphi (k) + \sum _{k \in S} \varphi (k) + \sum _{k \in \overline{S\,}} \varphi (k), \end{aligned}$$

where *S* is the set of $\lfloor \sqrt{n} \rfloor$-smooth integers in the interval $\big [ \lfloor \sqrt{n} + 1\rfloor , n \big ]$ and $\overline{S\,}$ is the set of integers that are not $\lfloor \sqrt{n} \rfloor$-smooth in the same interval. Two helper algorithms are introduced to process the smooth and the non-smooth integers, which are formally defined in one of the next sections. The overall algorithm still runs in *O*(*n*) time, but uses only $O(n^{1/2 + o(1)})$ memory.

Algorithm C is based on the following novel formula for the length of $F_n$:

5 $$\begin{aligned} |F_n| = \frac{(n+3) n}{2} - \sum _{k = 2}^{u(n)} \left| F_{\left\lfloor \frac {n}{k} \right\rfloor } \right| - \sum _{k = 1}^{\lfloor \sqrt{n} \rfloor } \left( \left\lfloor \frac{n}{k } \right\rfloor - \left\lfloor \frac{n}{k+1} \right\rfloor \right) \cdot | F_k |, \end{aligned}$$

where $u(n) = \big \lfloor n / \big ( \lfloor \sqrt{n} \rfloor + 1 \big ) \big \rfloor$. The formula works for all $n > 1$. Supplementary Sect. S6 proves this formula by splitting the sum in formula (2) at *u*(*n*) and then expressing the second sum in a different way. This leads to an algorithm that runs in $O(n^{3/4})$ time and uses $O(\sqrt{n})$ memory.

Algorithm D can be derived from formula (5) by splitting the first sum at $v(n) = \big \lfloor \frac {n}{\big (\lfloor \root 3 \of {n^2} \rfloor {+} 1 \big )} \big \rfloor$ as follows:

6 $$\begin{aligned} \sum _{k = 2}^{u(n)} \big | F_{\left\lfloor \frac {n}{k} \right\rfloor } \big |&= \sum _{k = 2}^{v(n)} \big | F_{\left\lfloor \frac {n}{k} \right\rfloor } \big | \, + \sum _{k = v(n) + 1}^{u(n)} \big | F_{\left\lfloor \frac {n}{k} \right\rfloor } \big |. \end{aligned}$$

Reversing the direction of the last sum and changing the index variable from *k* to *i* we get:

7 $$\begin{aligned} \sum _{k = 2}^{u(n)} \big | F_{\left\lfloor \frac {n}{k} \right\rfloor } \big |&= \sum _{k = 2}^{v(n)} \big | F_{\left\lfloor \frac {n}{k} \right\rfloor } \big | \,+\, \sum _{i = 0}^{w(n)} \big | F_{\left\lfloor \frac{n}{u(n) - i} \right\rfloor } \big |, \end{aligned}$$

where $w(n) = u(n) - v(n) - 1$. To continue the derivation, let $b_i$ be the order of the Farey sequence in the last sum in formula (7), i.e., $b_i = {\textstyle \big \lfloor \frac{n}{u(n) - i} \big \rfloor }$. Then,

8 $$\begin{aligned} \sum _{i = 0}^{w(n)} \big | F_{\left\lfloor \frac{n}{u(n) - i} \right\rfloor } \big | = \sum _{i = 0}^{w(n)} \big | F_{b_i} \big |. \end{aligned}$$

This sum can be expressed using Euler’s totients for all integers in the intervals $[a_0, b_0], \, [a_1, b_1], \dotsc , [a_{w(n)}, b_{w(n)}]$, where $a_0 = \lfloor \sqrt{n} \rfloor + 1$, $b_0 = {\textstyle \big \lfloor \frac{n}{u(n)} \big \rfloor }$, and $a_i = b_{i - 1} + 1$ for $i \in \{1, 2, \dotsc , w(n)\}$. To derive this result, the value of $\big | F_{b_i} \big |$ can be expressed as the value of $\big | F_{b_{i-1}} \big |$ plus the sum of Euler’s totients for all integers in the interval $[a_i, b_i]$. That is,

9 $$\begin{aligned} \big | F_{b_i} \big |&= \big | F_{\lfloor \sqrt{n} \rfloor } \big | + \varphi ( \underbrace{\lfloor \sqrt{n} \rfloor + 1}_{a_0} ) + \varphi (\lfloor \sqrt{n} \rfloor + 2) + \cdots + \varphi (b_i - 1) + \varphi (b_i) \nonumber \\&= \underbrace{\big | F_{\lfloor \sqrt{n} \rfloor } \big | + \sum _{m = a_0}^{b_0} \varphi (m) + \sum _{m = a_1}^{b_1} \varphi (m) + \cdots + \sum _{m = a_{i-1}}^{b_{i-1}} \varphi (m) }_{\displaystyle \big | F_{b_{i - 1}} \big |} + \sum _{m = a_i}^{b_i} \varphi (m) \nonumber \\&= \big | F_{b_{i - 1}} \big | + \sum _{m = a_i}^{b_i} \varphi (m). \end{aligned}$$

Therefore, formula (8) can be expressed as follows:

10 $$\begin{aligned} \sum _{i = 0}^{w(n)} \big | F_{\left\lfloor \frac{n}{u(n) - i} \right\rfloor } \big | = \sum _{i = 0}^{w(n)} \left( \big | F_{b_{i - 1}} \big | + \sum _{m = a_i}^{b_i} \varphi (m) \right) . \end{aligned}$$

Plugging the right-hand side of the last equation into formula (6) and that result into (5) leads to the following formula:

11 $$\begin{aligned} |F_n| = \frac{(n+3) n}{2} - \sum _{k = 2}^{v(n)} \left| F_{\left\lfloor \frac {n}{k} \right\rfloor } \right| - \sum _{i = 0}^{w(n)} \underbrace{\bigg ( \big | F_{b_{i - 1}} \big | + \sum _{m = a_i}^{b_i} \varphi (m) \bigg )}_{\displaystyle \big | F_{b_i} \big |} - \sum _{k = 1}^{\lfloor \sqrt{n} \rfloor } \left( \left\lfloor \frac{n}{k } \right\rfloor - \left\lfloor \frac{n}{k+1} \right\rfloor \right) \cdot \underbrace{\left( 1 + \sum _{m=1}^k \varphi (m) \right) }_{\displaystyle | F_k |}, \end{aligned}$$

where $w(n) = u(n) - v(n) - 1$, $u(n) = \big \lfloor n / \big ( \lfloor \sqrt{n} \rfloor + 1 \big ) \big \rfloor$, and $v(n) = \big \lfloor \frac {n}{\big (\lfloor \root 3 \of {n^2} \rfloor {+} 1 \big )} \big \rfloor$. Also, $a_0 = \lfloor \sqrt{n} \rfloor + 1$ and $b_0 = {\textstyle \big \lfloor \frac{n}{u(n)} \big \rfloor }$. Furthermore, $a_i = b_{i - 1} + 1$ and $b_i = {\textstyle \big \lfloor \frac{n}{u(n) - i} \big \rfloor }$ for $i \in \{1, 2, \dotsc , w(n) \}$. The last term in (11) expands the value of $|F_k|$ as a sum of totients, which suggests how it can be computed iteratively. Because the index *k* in the last sum in (11) goes up to $\lfloor \sqrt{n} \rfloor$, the last value of $|F_k|$ is equal to $| F_{\lfloor \sqrt{n} \rfloor } |$. If the last sum is processed first, then the computed value of $| F_{\lfloor \sqrt{n} \rfloor } |$ can be used to bootstrap the calculation of $| F_{b_i} |$ in the middle sum, e.g., see formula (9). These insights lead to algorithm D, which runs in $O(n^{2/3})$ time and uses $O(n^{2/3})$ memory.

Algorithm E was inspired by a modified version of formula (11) that is shown below:

12 $$\begin{aligned} |F_n| = \frac{(n+3) n}{2} - \sum _{k = 2}^{v(n)} \left| F_{\left\lfloor \frac {n}{k} \right\rfloor } \right| - \! \sum _{i = 0}^{w(n)} \! \bigg ( \big | F_{b_{i - 1}} \big | + \underbrace{\sum _{{m \in S_i}} \varphi (m) + \sum _{{m \in \overline{S_i}}} \varphi (m) }_{\displaystyle B[i]} \bigg ) \! - \! \sum _{k = 1}^{\lfloor \sqrt{n} \rfloor } \!\! \left( \left\lfloor \frac{n}{k } \right\rfloor - \left\lfloor \frac{n}{k+1} \right\rfloor \right) \!\cdot \! \underbrace{\left( 1 + \sum _{m=1}^k \varphi (m) \right) }_{\displaystyle | F_k |} \!. \end{aligned}$$

In other words, this modification splits one of the sums in (11) as follows:

13 $$\begin{aligned} \sum _{m = a_i}^{b_i} \varphi (m) = \underbrace{\sum _{m \in S_i} \varphi (m) + \sum _{m \in \overline{S_i}} \varphi (m)}_{B[i]}, \end{aligned}$$

where $S_i$ is the set of $\lfloor \sqrt{n} \rfloor$-smooth integers in the interval $I_i = [a_i, b_i]$ and $\overline{S_i}$ is the set of integers that are not $\lfloor \sqrt{n} \rfloor$-smooth in the same interval. Thus, algorithm E uses a similar approach to summing the totients as in formula (4) that is used by algorithm B. In this case, however, the smooth and the non-smooth numbers are processed separately but the values of their corresponding totients are accumulated in the array *B*. This change is sufficient to reduce the memory complexity from $O(n^{2/3})$ to $O(\sqrt{n})$. The time complexity remains unchanged, i.e., $O(n^{2/3})$.

## Related work

A prime sieve is an efficient algorithm for generating all prime numbers in some interval^16^. The sieve of Eratosthenes is probably the oldest and the most well-known prime sieve algorithm^4,16,17^. It can generate all prime numbers in the interval [1, *n*] in $O(n \log \log n)$ time. Our algorithms, however, do not use the sieve of Eratosthenes. Instead, they use the linear sieve^18^ or the sieve of Atkin^19^. These two sieves do not have the factor $\log \log n$ in their time complexity, i.e., they run in *O*(*n*) time.

Algorithms A, B, D, and E use the linear sieve algorithm^18^, making it possible to compute Euler’s totients for all integers between 1 and *n* in *O*(*n*) time. In addition to the linear sieve, Algorithms B and E also use the sieve of Atkin^19^ to reduce the space complexity. Algorithm C does not use a sieve.

The sieve of Atkin generates all prime numbers between 1 and *N* in *O*(*N*) time^19^. It uses $O(N^{1/2 + o(1)})$ memory^19^. Algorithm E uses this sieve with $N = \beta \in O(n^{2/3})$ to enumerate numbers that are not $(\alpha -1)$-smooth in the interval $[\alpha , \beta ]$. This sub-routine and other optimizations reduce its space complexity down to $O(\sqrt{n} )$. This makes it practical to compute $|F_n|$ for very large values of *n* by using the computer’s memory more efficiently. Algorithm E enumerates the smooth numbers in $[\alpha , \beta ]$ separately from the non-smooth numbers, which are complementary to them. Smooth numbers^20–22^ are often used in computational number theory for primality testing, integer factorization, and computing discrete logarithms.

Computing the length of the Farey sequence is related to several other problems. For example, by subtracting 1 from $|F_n|$ one can obtain^12^ the sum of all Euler’s totients between 1 and *n*. Some authors have described approaches for attacking the order statistics problem^23,24^ and the rank problem^25^ on Farey sequences. Their work is related to methods for summing the values of the Möbius function^26^, which is equivalent to computing the Mertens function. The time complexity class of these methods is $O(n^{2/3} (\log \log n)^{1/3})$. More recently, the best time complexity for computing the Mertens function was estimated^27^ at $O(n^{2/3 + \varepsilon })$, i.e., slightly worse than $O(n^{2/3})$. The time complexity of our most efficient algorithm for computing $|F_n|$ is exactly $O(n^{2/3})$, i.e., without any additional small factors that depend on *n*. Its space complexity is $O(\sqrt{n})$. All estimates use the same computational model, which assumes that any arithmetic or storage operation on any integer runs in *O*(1) time and storing any integer requires *O*(1) memory.

In digital signal processing, the Inverse Chirp Z-Transform (ICZT) is a generalization^6^ of the Inverse Fast Fourier Transform (IFFT). This transform is parametrized by the complex numbers *A* and *W*. They define a logarithmic spiral contour formed by the sampling points $A W^{-k}$ where $k \in \{0, 1, 2, \dotsc , n - 1\}$ and *n* is the size of the transform. For the special case when the magnitudes of both *A* and *W* are equal to 1, the contour is restricted to lie on the unit circle in the complex plane. In this case, the ICZT has a singularity^5^ if and only if the polar angle of *W* can be expressed as $\frac {2 \pi p}{q}$ where $\frac {p}{q}$ is an element of $F_{n-1}$. Consequently, the numerical error profile for the ICZT of size *n* is determined^5^ by the elements of $F_{n-1}$. Therefore, the number of possible values of the parameter *W* for which the transform is singular is equal to the length of $F_{n-1}$.

## Algorithm A

Algorithm 1 shows the pseudo-code for the first algorithm that computes $|F_n|$ without enumerating the sequence elements. On line 2 the algorithm uses a linear sieve to compute a list *P* of all prime numbers in the interval [1, *n*]. The linear sieve also returns an array $L_p$ of size *n* such that its *k*-th element $L_p[k]$ is equal to the smallest prime factor of *k*. On line 3 the elements of $L_p$ are used to compute an array $\varphi$ that contains the values of Euler’s totient function $\varphi (1), \varphi (2), \dotsc , \varphi (n)$. The rest of the code (i.e., lines 4–7) uses formula (3) to compute the value of $|F_n|$, i.e., it sums up the *n* totients and adds 1 to the sum *s*. 
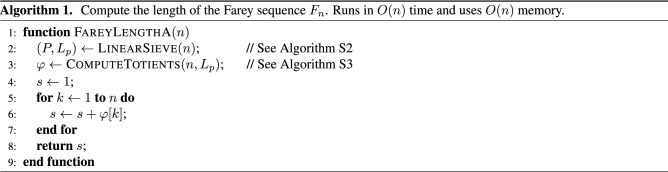


Supplementary Section S3 gives the pseudo-code for the linear sieve algorithm and for the function ComputeTotients. The appendix also proves a property of Euler’s totient function that makes it possible to compute $\varphi (1), \varphi (2), \dotsc , \varphi (n)$ from the array $L_p$ in *O*(*n*) time. Thus, the computational complexity of Algorithm 1 is *O*(*n*). It uses *O*(*n*) memory.

This algorithm is fairly easy to describe, but it is not very fast. It also uses a lot of memory. It is important to understand how it works, however, as some of the more efficient algorithms use the same approach to solve a part of the problem.

## Algorithm B

This section describes another algorithm for computing $|F_n|$ that also runs in *O*(*n*) time, but uses only slightly more than $O(\sqrt{n})$ memory. The main idea is to split all integers in the interval $[ \lfloor \sqrt{n} \rfloor + 1, n ]$ into two disjoint sets, *S* and $\overline{S\,}$, such that their elements and their corresponding Euler’s totients can be computed using $O(\sqrt{n})$ memory instead of *O*(*n*). The set *S* is the set of $\lfloor \sqrt{n} \rfloor$-smooth numbers and the set $\overline{S\,}$ is the set of numbers that are not $\lfloor \sqrt{n} \rfloor$-smooth.

Both smooth and non-smooth numbers are integers. They are defined as follows:

- A positive integer *N* is *B*-smooth if its largest prime factor *p* does not exceed *B*, i.e., $p \le B$.
- A positive integer *N* is not *B*-smooth if its largest prime factor *p* is strictly greater than *B*, i.e., $p > B$.

For example, the number 100 is 5-smooth because $100 = 5^2 \cdot 2^2$. It is also 6-smooth, 7-smooth, 8-smooth, etc. But it is not *k*-smooth for $k \in \{2, 3, 4 \}$. To give another example, the number 84 is 7-smooth because $84 = 7 \cdot 3 \cdot 2^2$. It is also 8-smooth, 9-smooth, etc. But it is not 5-smooth because its largest prime factor is 7, which is greater than 5. In fact, it is not *k*-smooth for $k \in \{2, 3, 4, 5, 6\}$.

Algorithm 2 starts by computing $r = \lfloor \sqrt{n} \rfloor$ exactly using the method^28^ described in Supplementary Sect. S7. Next, it implements the summation of Euler’s totients in a way that avoids allocating an array of length *n* to store them. In fact, the algorithm uses only slightly more than $O(\sqrt{n})$ memory. This is achieved by running the linear sieve only for $k \in \{1, 2, \dotsc , \lfloor \sqrt{n} \rfloor \}$. Starting from $\lfloor \sqrt{n} + 1\rfloor$, the sum in formula (3) is split into three separate sums as follows:

14 $$\begin{aligned} |F_n| = 1 + \sum _{k = 1}^{\lfloor \sqrt{n} \rfloor } \varphi (k) + \sum _{k \in S} \varphi (k) + \sum _{k \in \overline{S\,}} \varphi (k), \end{aligned}$$

where *S* is the set of $\lfloor \sqrt{n} \rfloor$-smooth numbers in the interval $\big [ \lfloor \sqrt{n} + 1\rfloor , n \big ]$ and $\overline{S\,}$ is the set of numbers that are not $\lfloor \sqrt{n} \rfloor$-smooth in the same interval. A similar technique is used in Algorithm 8 to reduce its space complexity from $O(n^{2/3})$ to $O(\sqrt{n})$. 
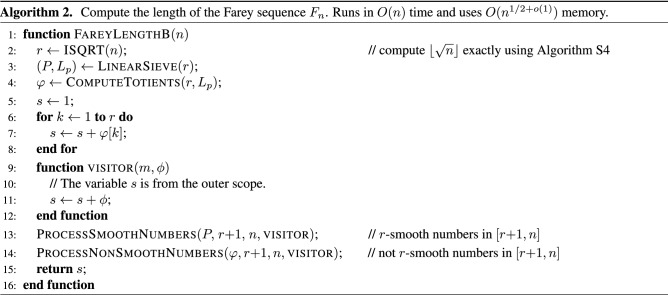


The smooth numbers are processed with Algorithm 3. It enumerates the $(\alpha - 1)$-smooth numbers in the interval $[\alpha , \beta ]$. More specifically, the algorithm implements a depth-first traversal of the search space formed by all integers in this interval that have prime factorizations that include only prime factors from the array *P*, which contains the prime numbers in the interval $[1, \alpha - 1]$. The algorithm calls the visitor function for each $(\alpha - 1)$-smooth number *m* in the interval $[\alpha , \beta ]$ and its Euler’s totient, which is computed using formula (22) that is described in Supplementary Sect. S3. Algorithm 3 runs in $O(\beta )$ time and uses $O(\log \beta )$ memory. It traverses no more than $2 \beta$ values of *m* and performs *O*(1) operations for each of them. The space complexity of this algorithm is determined by the maximum depth of the stack, which cannot exceed $\lfloor \log _2 \beta \rfloor$ because 2 is the smallest possible prime factor in the list *P*.

The non-smooth numbers are handled by Algorithm 4. It traverses the numbers that are not $(\alpha -1)$-smooth in the interval $[\alpha , \beta ]$ and calls the visitor function for each non-smooth number and for all its integer multiples that fit in the interval. The computational complexity of this algorithm is determined by the sieve of Atkin, which runs in $O(\beta )$ time and uses $O(\beta ^{1/2 + o(1)})$ memory^19^. In other words, Algorithm 4 performs *O*(1) operations for each non-smooth number that it visits, which doesn’t change its time complexity class. Thus, the algorithm runs in $O(\beta )$ time and uses $O(\beta ^{1/2 + o(1)})$ memory. 
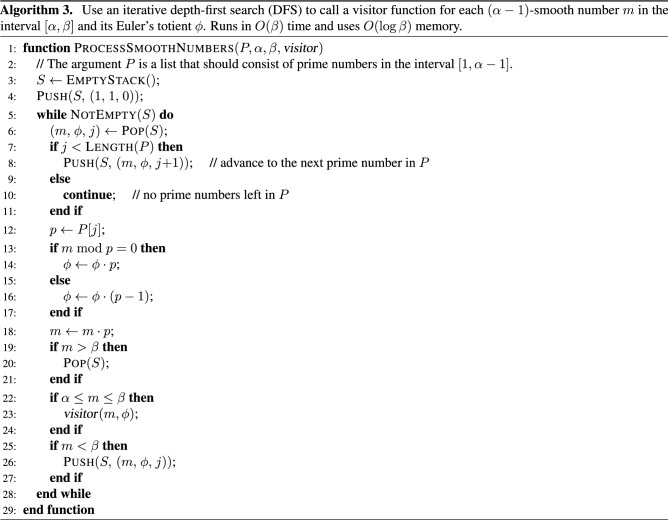

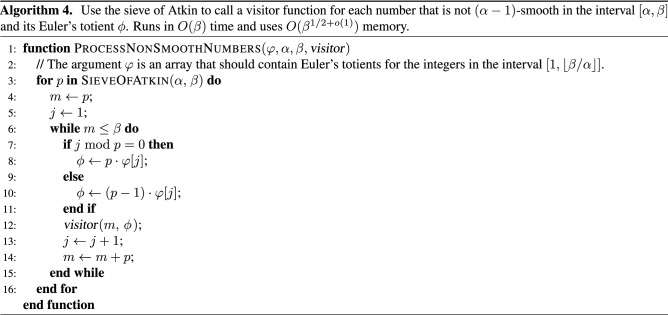




**Figure 2:**
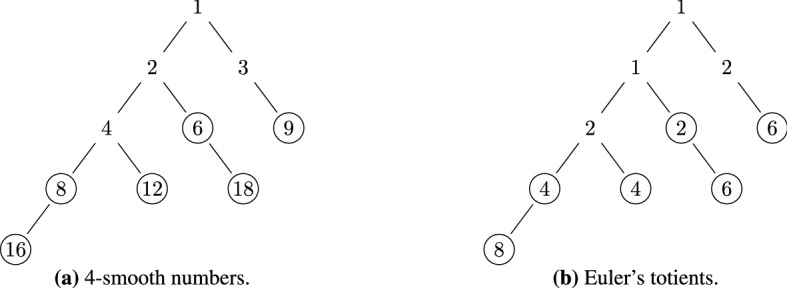
Traversal of the 4-smooth numbers in the interval [5, 20] by Algorithm 3 when $n = 20$. The tree of $\lfloor \sqrt{n} \rfloor$-smooth numbers is shown in (**a**). Their corresponding totients are shown in (**b**). The numbers for which the visitor function is called are circled. The list of prime numbers in this case is $P = (2, 3)$. Thus, each left branch in (**a**) multiplies the parent node by 2 and each right branch multiplies it by 3. If the product exceeds 20, then the corresponding branch or leaf is excluded from the tree.

**Figure 3:**
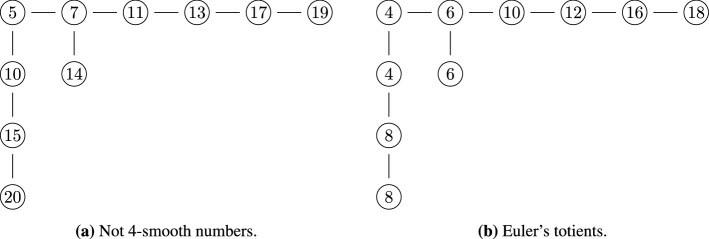
Enumeration of not 4-smooth numbers in the interval [5, 20] by Algorithm 4 when $n = 20$. The tree of non-smooth numbers is shown in (**a**), their corresponding totients are shown in (**b**). The visitor function is called for all circled numbers.

To give a concrete example, let $n = 20$. Figure 2a shows the search tree for the $\lfloor \sqrt{n} \rfloor$-smooth numbers in the interval [5, 20]. Algorithm 3 calls the visitor function for the pairs $(k, \varphi (k))$ where *k* is 4-smooth (i.e., $\lfloor \sqrt{20} \rfloor = 4$) and $\varphi (k)$ is its totient. The totients are shown in Fig. 2b. The algorithm calls the visitor function only for $k \ge \lfloor \sqrt{20} + 1 \rfloor = 5$, i.e., only for the circled integers and totients shown in the figure. The tree in this case is binary because the list $P = (2, 3)$ contains only two prime numbers. Thus, all left branches multiply the parent node by 2 and all right branches multiply it by 3. The order of enumeration is: 1, 2, 4, 8, 16, 12, 6, 18, 3, 9 (i.e., pre-order traversal).

Figure 3 visualizes the enumeration of the numbers that are not $\lfloor \sqrt{n} \rfloor$-smooth for $n = 20$. Algorithm 4 calls the sieve of Atkin to generate the prime numbers in the interval [5, 20], i.e., 5, 7, 11, 13, 17, 19. This is done one-at-a-time, i.e., without storing the list of primes in memory. The algorithm also calls the visitor function for each pair $(k, \varphi (k))$, where *k* is each prime number or its integer multiples that fit in the interval. The enumeration order here is: 5, 10, 15, 20, 7, 14, 11, 13, 17, and 19.

**Table 1:** Values of Euler’s totient function $\varphi (k)$ for all integers *k* between 1 and 20.

*k*	1	2	3	4	5	6	7	8	9	10	11	12	13	14	15	16	17	18	19	20
$\varphi (k)$	1	1	2	2	4	2	6	4	6	4	10	4	12	6	8	8	16	6	18	8

Table 1 shows the values of Euler’s totient function $\varphi (k)$ for each *k* between 1 and 20. The sum of these totients is equal to 128. Therefore, $|F_{20}| = 1 + 128 = 129$. To compute this result, Algorithm 2 first adds $1 + \varphi (1) + \varphi (2) + \varphi (3) + \varphi (4) = 7$. Then, it calls Algorithm 3, which computes the sum of all circled numbers in Fig. 2b (i.e., $4 + 8 + 4 + 2 + 6 + 6 = 30$) and adds it to 7. Finally, it calls Algorithm 4, which computes the circled numbers in Fig. 3b and adds their sum (i.e., 92) to the previous result. Thus, the return value for $|F_{20}|$ is $7 + 30 + 92 = 129$.

## Algorithm C

This section describes another algorithm for computing $|F_n|$. Unlike algorithms A and B, it does not sum Euler’s totients. Instead, it uses formula (5), which expresses $|F_n|$ in terms of values of $|F_k|$ for $k < n$. The algorithm uses this formula multiple times and stores the computed values for $|F_k|$ in a lookup table, which is described in Supplementary Sect. S8.

Algorithm 5 gives the pseudo-code for algorithm C. First, the code sets the variables *r* and *u* to the upper limits of the two summations in formula (5), which are $\lfloor \sqrt{n} \rfloor$ and *u*(*n*), respectively. Next, it creates an empty lookup table and initializes its first entry, which corresponds to $|F_1|=2$. Then, the two for-loops fill the required entries in the lookup table in an order that makes it possible to eventually compute $|F_n|$. The first for-loop computes $|F_2|, |F_3|, |F_4|, \dotsc , |F_{\lfloor \sqrt{n} \rfloor }|$ in that order. The second for-loop computes $|F_{\lfloor n/u(n) \rfloor }|, |F_{\lfloor n / (u(n)-1) \rfloor }|, |F_{\lfloor n / (u(n)-2) \rfloor }|, \dotsc , |F_{\lfloor n / 2 \rfloor }|, | F_{\lfloor n / 1 \rfloor }|$. The value of $|F_n|$ is computed in the last iteration when the value of the variable *j* is equal to 1.

Each iteration through line 7 or line 10 corresponds to an instance of formula (5). A helper function implements this computation. Its pseudo-code is listed in Algorithm 6. It has two for-loops that correspond to the two summations in formula (5). Each call to the helper function stores the computed value of $|F_m|$ in the lookup table so that it is available later on.

Supplementary Section S10 proves that the order in which the algorithm fills the lookup table entries is correct. It shows that computing each subsequent entry requires accessing only those lookup table elements that have already been set in previous iterations. The appendix also proves that Algorithm 5 runs in $O(n^{3/4})$ time and uses $O(\sqrt{n})$ memory. Supplementary Section S11 gives a recursive version of this algorithm. 
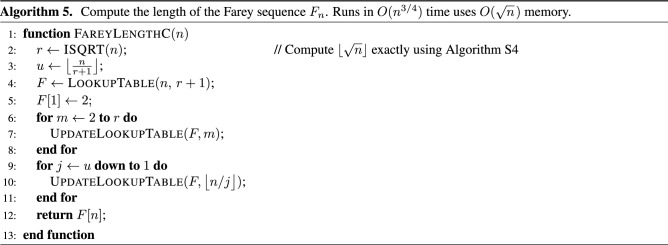

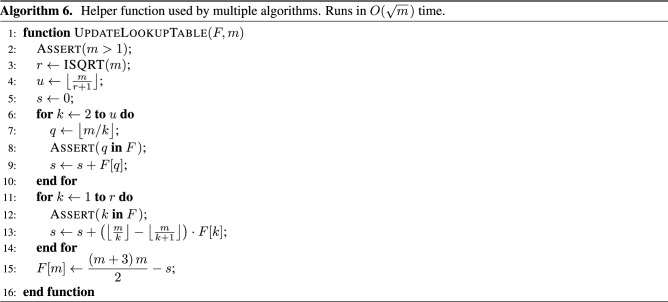




**Figure 4:**
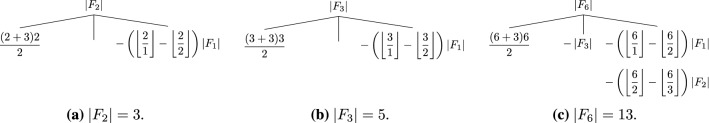
Visualizations of the three instances of formula (5) in the execution of algorithm C when it is called with $n = 6$. In this case, the first for-loop of the algorithm invokes the formula to compute $|F_2|$ as shown in (**a**). The second for-loop computes $|F_3|$ and $|F_6|$. The trees for these two terms are shown in (**b**) and (**c**), respectively. The result is $|F_6| = 13$.

Figure 4 visualizes the computation performed by algorithm C (i.e., Algorithm 5) for $n = 6$. This process uses formula (5) three times. Each tree shown in this figure corresponds to one call to the helper function. The root node is the term $|F_m|$ that the helper function stores in the lookup table. The three branches show the terms in formula (5) that the function adds to compute the value of $|F_m|$. The first for-loop in algorithm C computes $|F_2|$ and stores its value in the lookup table. Figure 4a visualizes the corresponding instance of formula (5) in which the middle branch is empty. The second for-loop computes the values of $|F_3|$ and $|F_6|$. The corresponding trees are shown in Fig. 4b and c, respectively. Note that there is no tree for $|F_1|$ because the algorithm stores its value in the lookup table during initialization, before the first for-loop. Also, note that the tree for $|F_6|$ uses the previously computed values for $|F_2|$ and $|F_3|$.

Supplementary Section S10 proves that for each $m > 1$, every term $|F_m|$ that appears in a branch of some tree also appears as a root node for one of the preceding trees. In other words, the order in which the algorithm fills the lookup table is correct.

## Algorithm D

This section describes the fourth algorithm for computing $|F_n|$. Algorithm D can be viewed as an optimized version of algorithm C that runs faster, i.e., $O(n^{2/3})$ instead of $O(n^{3/4})$ time. This run-time improvement, however, is achieved at the expense of using more memory than algorithm C, i.e., $O(n^{2/3})$ instead of $O(\sqrt{n})$ memory. The pseudo-code is given in Algorithm 7.

The first for-loop in algorithm C computes the values of $|F_1|, |F_2|, \dotsc , |F_{\lfloor \sqrt{n} \rfloor }|$ using the helper function, which implements formula (5). This process requires $O(n^{3/4})$ time. Because the orders of these Farey sequences form a contiguous range of integers, however, we can compute their lengths a bit faster. That is, they can be computed in $O(\sqrt{n})$ time by adding Euler’s totients $\varphi (1), \varphi (2), \dotsc , \varphi (\lfloor \sqrt{n} \rfloor )$. These totients, in turn, can be generated in $O(\sqrt{n})$ time using Algorithms S2 and S3.

Thus, the computation performed by the first for-loop in algorithm C can be implemented so that it runs in $O(\sqrt{n})$ instead of $O(n^{3/4})$ time. This optimization alone won’t affect the overall computational complexity because the second for-loop would still run in $O(n^{3/4})$ time. It cannot be optimized in the same way as the first for-loop because the orders of the Farey sequences $F_{\lfloor n/u(n) \rfloor }, F_{\lfloor n/(u(n) - 1) \rfloor }, \dotsc , F_{\lfloor n/2 \rfloor }, F_n$ do not form a contiguous range of integers.

In addition to the optimization described above, it is also possible to change the split point between the two stages of computation so that the first stage computes more than $\lfloor \sqrt{n} \rfloor$ entries of the lookup table. That is, instead of running the sieve-based approach up to $\lfloor \sqrt{n} \rfloor$, we can let it run for some number of additional iterations beyond that. Correspondingly, the starting value of the loop variable *j* in the second for-loop can be adjusted to be smaller than $u(n) = \big \lfloor n / (\lfloor \sqrt{n} \rfloor + 1) \big \rfloor$. Supplementary Section S12 shows that the optimal running time is achieved when the first stage runs up to $\lfloor n^{2/3} \rfloor$ and when the last for-loop starts from $v(n) = \big \lfloor n / (\lfloor n^{2/3} \rfloor + 1) \big \rfloor$ instead of *u*(*n*).

Algorithm D is the result of making these changes to algorithm C. The loop on lines 10–14 computes $|F_1|, |F_2|, \dotsc , |F_{\lfloor \sqrt{n} \rfloor }|$ by using the linear sieve to generate an array of totients and then summing them similarly to what algorithm A does. The for-loop on lines 15–23 implements formula (10) to compute $\big | F_{\left\lfloor \frac{n}{u(n)} \right\rfloor } \big |, \big | F_{\left\lfloor \frac{n}{u(n)-1} \right\rfloor } \big |, \dotsc , \big | F_{\left\lfloor \frac{n}{u(n)-w(n)} \right\rfloor } \big |$ by adding the totients from the array $\varphi$. The last value is equivalent to $\big | F_{\left\lfloor \frac{n}{v(n) + 1} \right\rfloor } \big |$ because $w(n) = u(n) - v(n) - 1$. The last for-loop (i.e., lines 24–26) computes $\big | F_{\left\lfloor \frac{n}{v(n)} \right\rfloor } \big |, \big | F_{\left\lfloor \frac{n}{v(n) - 1} \right\rfloor } \big |, \dotsc , \big | F_{\left\lfloor \frac{n}{2} \right\rfloor } \big |, \big | F_n \big |$ using formula (5) that is implemented by the helper function.

Supplementary Appendix S13 gives an alternative, shorter version of this algorithm in which the second for-loop is removed (i.e., lines 15–23). In that version, the first for-loop runs up to $m = c$, instead of $m = r$. This fills more entries in the lookup table than necessary for computing $|F_n|$, but the space complexity remains in $O(n^{2/3})$. The time complexity also remains in $O(n^{2/3})$. Algorithm 7 was selected for presentation in the main paper because it is easier to map it to the formulas and because in this form it is easier to understand the optimization performed by algorithm E, which is discussed next. 
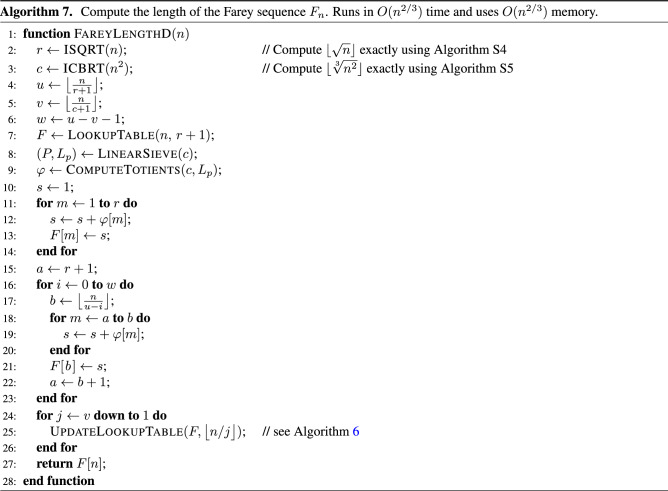


## Algorithm E

This section describes algorithm E, which computes the length of a Farey sequence of order *n*. Its pseudo-code is shown in Algorithm 8. Its time complexity is $O(n^{2/3})$, which is the same as that of algorithm D. However, algorithm E uses only $O(\sqrt{n})$ memory. This improvement makes it the most efficient algorithm for computing $|F_n|$ described in this paper.

Algorithm E can be viewed as a modified version of algorithm D. It changes how the entries of the lookup table are computed for indices that fall in the interval $[\alpha , \beta ]$, where $\alpha = \lfloor \sqrt{n} \rfloor + 1$ and $\beta = \lfloor n / (v(n) + 1) \rfloor$. The process of computing all other entries of the lookup table is similar for both algorithms. That is, both algorithms use the linear sieve to compute the totients $\varphi (1), \varphi (2), \dotsc , \varphi (\lfloor \sqrt{n} \rfloor )$ and then add them to compute $|F_1|, |F_2|, \dotsc , |F_{\lfloor \sqrt{n} \rfloor }|$. Also, both algorithms use the helper function to compute the entries of the lookup table for indices that fall in the interval $[\beta + 1, n]$, including *n*.

For indices in the interval $[\alpha , \beta ]$, algorithm D uses the linear sieve. Because the value of $\beta$ is in $O(n^{2/3})$, its memory complexity is $O(n^{2/3})$. In contrast, algorithm E uses the linear sieve only in the interval $[1, \alpha - 1]$ and $\alpha = \lfloor \sqrt{n} \rfloor$ is in $O(\sqrt{n})$. For each integer *m* in $[\alpha , \beta ]$, algorithm E calls the visitor function with arguments *m* and $\varphi (m)$. More specifically, Algorithm 3 is used to process all $\lfloor \sqrt{n} \rfloor$-smooth numbers in that interval and Algorithm 4 is called to process all numbers that are not $\lfloor \sqrt{n} \rfloor$-smooth in the same interval. Algorithms 3 and 4 use only $O(\sqrt{n})$ memory because $\beta$ is in $O(n^{2/3})$.

The visitor function determines the index *i* of the corresponding interval $I_i = [a_i, b_i]$ in formula (13) where *m* falls and then adds $\varphi (m)$ to the array element *B*[*i*]. After processing all integers in $[\alpha , \beta ]$, the element *B*[*i*] becomes equal to the sum of Euler’s totients for all integers in $I_i$. Then, the algorithm computes the values of $|F_{b_i}|$ by adding the elements of the array *B* to $\big | F_{\lfloor \sqrt{n} \rfloor } \big |$. The length of the array *B* is equal to $w(n) + 1$, which is in $O(\sqrt{n})$. This reduces the amount of memory required to process all integers in the interval $[\alpha , \beta ]$. Therefore, the space complexity of algorithm E is $O(\sqrt{n})$. Supplementary Section S14 gives a formal proof for its time and space complexity. 
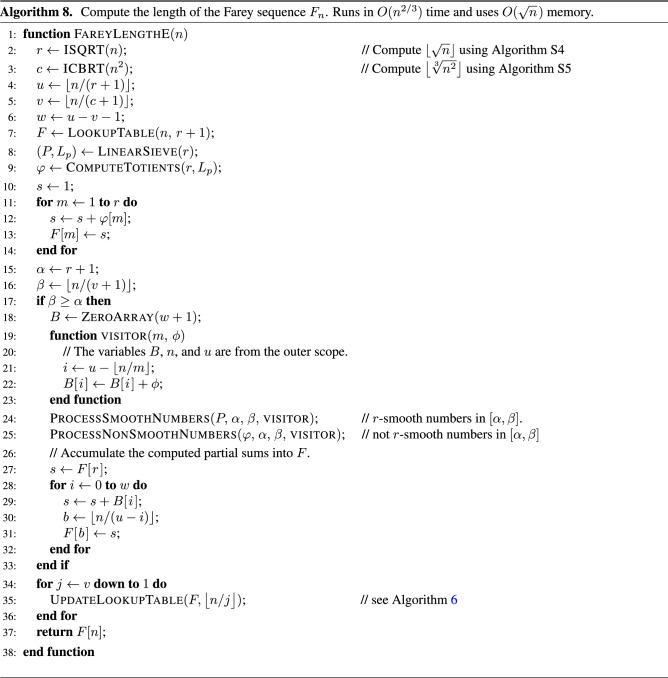


To give a concrete example, let’s look at how algorithm E computes $|F_n|$ for $n = 120$. In this case, $\lfloor \sqrt{n} \rfloor = \lfloor \sqrt{120} \rfloor = 10$. First, on lines 8–9 the algorithm uses the linear sieve to compute the values of $\varphi (1), \varphi (2), \dotsc , \varphi (\lfloor \sqrt{n} \rfloor )$. Supplementary Section S3 describes this process and gives the pseudo-code for the two algorithms that implement it. Second, the loop on lines 10–14 adds these totients to compute the values of $|F_1|, |F_2|, \dotsc , |F_{10}|$. Essentially, this is an implementation of formula (1).

**Figure 5:**
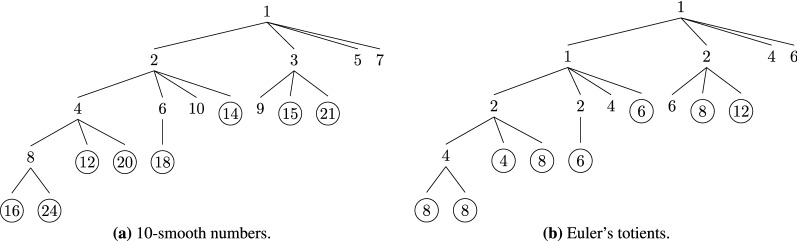
Enumeration of 10-smooth numbers and their Euler’s totients in the interval [11, 24].

Next, on line 24, the algorithm processes the smooth numbers by invoking Algorithm 3. It calls a visitor function for each 10-smooth number in the interval $[\alpha , \beta ]$, which in this case is equal to [11, 24]. Figure 5 visualizes this process using two trees. The circled nodes in Fig. 5a show the 10-smooth numbers that the algorithm visits; the circled nodes in Fig. 5b show their Euler’s totients. The nodes that are not circled correspond to the 10-smooth numbers that are less than 11 and for which the visitor function is not called in Algorithm 3. The Euler’s totients shown in Fig. 5b are computed using formula (22). The algorithm visits the numbers in lexicographic order with respect to their prime factorizations, i.e., $2 \cdot 2 \cdot 2 \cdot 2 = 16$, $2 \cdot 2 \cdot 2 \cdot 3 = 24$, $2 \cdot 2 \cdot 3 = 12$, $2 \cdot 2 \cdot 5 = 20$, $2 \cdot 3 \cdot 3 = 18$, $2 \cdot 7 = 14$, $3 \cdot 5 = 15$, and $3 \cdot 7 = 21$.

**Figure 6:**
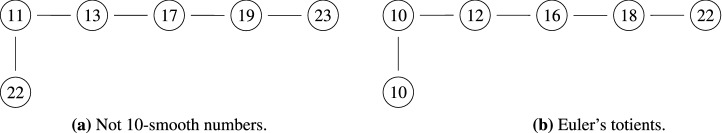
Enumeration of numbers that are not 10-smooth and their Euler’s totients in the interval [11, 24].

On line 25, the algorithm processes the non-smooth numbers by calling Algorithm 4, which visits the numbers that are not 10-smooth in the interval [11, 24] and their Euler’s totients. Figure 6 visualizes this process. Algorithm 4 uses the sieve of Atkin to generate the prime numbers in this interval, i.e., 11, 13, 17, and 23. For each of them the algorithm iterates over their integer multiples that fit in the interval. In this example, this results in visiting $11 \cdot 2 = 22$ after 11, but before 13. Figure 6b shows the totients for each of the non-smooth numbers in Fig. 6a. Their values are computed using formula (22).

The visitor function adds each circled totient from Figs. 5b and 6b to the corresponding element of the array *B*. After visiting all integers in [11, 24], the elements of *B* become equal to the sums of Euler’s totients in the corresponding sub-interval. Then, the algorithm sums them with $|F_{10}|$ to compute $|F_{12}|$, $|F_{13}|$, $|F_{15}|$, $|F_{17}|$, $|F_{20}|$, and $|F_{24}|$. More formally,

$$\begin{aligned} I_0&= [11, 12]&B[0]&= \varphi (11) + \varphi (12) = 14&|F_{12}|&= |F_{10}| + B[0] = 33 + 14 = 47 \\ I_1&= [13, 13]&B[1]&= \varphi (13) = 12&|F_{13}|&= |F_{12}| + B[1] = 47 + 12 = 59 \\ I_2&= [14, 15]&B[2]&= \varphi (14) + \varphi (15) = 14&|F_{15}|&= |F_{13}| + B[2] = 59 + 14 = 73 \\ I_3&= [16, 17]&B[3]&= \varphi (16) + \varphi (17) = 24&|F_{17}|&= |F_{15}| + B[3] = 73 + 24 = 97 \\ I_4&= [18, 20]&B[4]&= \varphi (18) + \varphi (19) + \varphi (20) = 32&|F_{20}|&= |F_{17}| + B[4] = 97 + 32 = 129 \\ I_5&= [21, 24]&B[5]&= \varphi (21) + \varphi (22) + \varphi (23) + \varphi (24) = 52&|F_{24}|&= |F_{20}| + B[5] = 129 + 52 = 181 \end{aligned}$$

**Figure 7:**
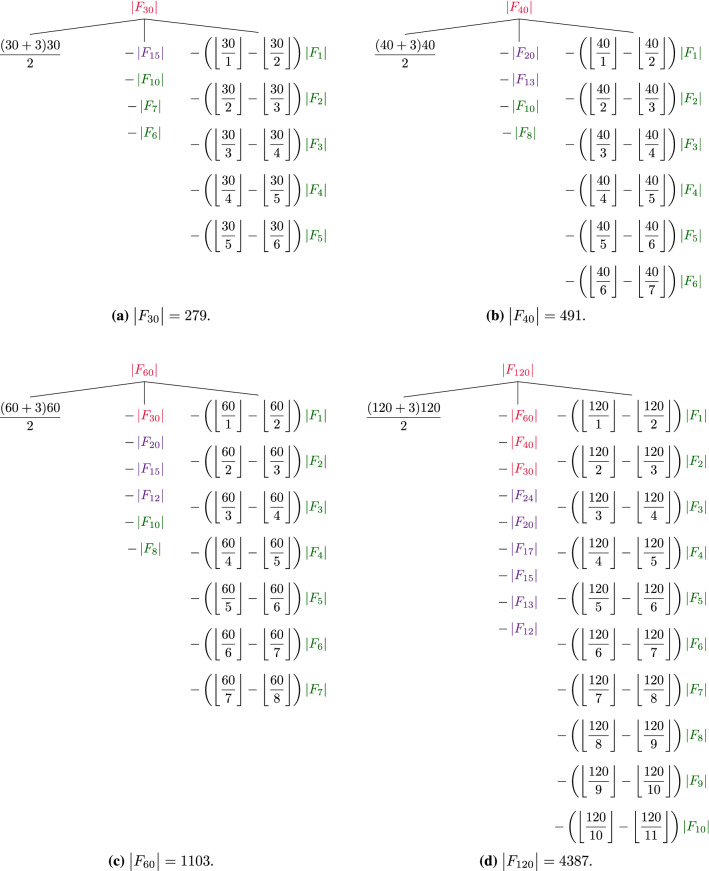
Visualization of the last stage of algorithm E in which the helper function is used to compute $|F_{30}|$, $|F_{40}|$, $|F_{60}|$, and $|F_{120}|$ when the algorithm is called with $n = 120$. The three colors correspond to the three different methods for computing intermediate results and storing them in the lookup table. That is, green terms are set on line 13. Purple terms are processed on line 31. Finally, the red terms are computed on line 35, during the call to UpdateLookupTable.

Finally, on lines 34–36, the algorithm computes $|F_{30}|$, $|F_{40}|$, $|F_{60}|$, and $|F_{120}|$ using the helper function as shown in Fig. 7. The colors in this figure indicate the three different methods that the algorithm uses to compute the lengths of Farey sequences and store them in the lookup table. That is, green corresponds to line 13, purple to line 31, and red to line 35.

## Results

We evaluated the performance of C and Python implementations of algorithms C, D, and E (i.e., Algorithms 5, 7, and 8). The value of *n* was varied between $10^0$ and $10^{14}$ in increments of 0.5 on the decimal logarithm scale. For each of the 29 values of *n*, each of the three algorithms, and each of the two implementations, the evaluation program ran the code 10 times while measuring its run time, memory usage, and the number of CPU instructions (see “Methods”). We also ran algorithm E for *n* between $10^{10}$ and $10^{18}$ and recorded the corresponding values of $|F_n|$.

**Figure 8:**
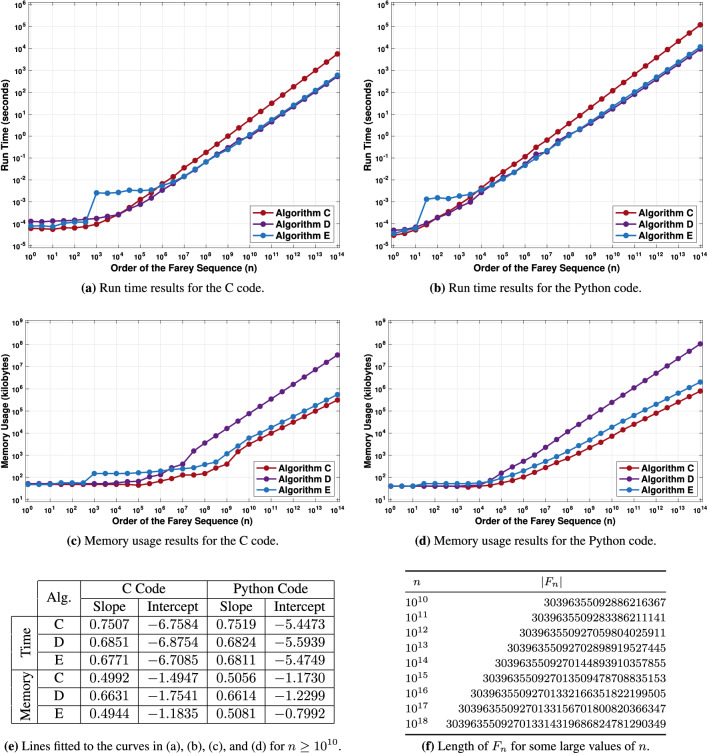
Evaluation results for C and Python implementations of algorithms C, D, and E. The run time curves plotted in (**a**) and the memory usage curves plotted in (**c**) are for the C code. The run time and memory usage plots for the Python code are shown in (**b**) and (**d**). The plots use the log scale for both axes and each point represents the average of 10 runs. The table in (**e**) gives the slopes and intercepts for lines that were fitted to the twelve curves in (**a**)–(**d**) in the region between $n = 10^{10}$ and $n = 10^{14}$. The table in (**f**) shows the lengths of $F_n$ computed with algorithm E for several large values of *n*.

Figure 8 visualizes the results of the experiments. Fig. 8a,b give the run time plots for C and Python, respectively. Fig. 8c,d show the corresponding memory usage plots. All of them are on a log scale. The vertical coordinate of each point is obtained by averaging the results of the 10 code runs. Figure 8e lists the slopes and intercepts for lines fitted to each of the 12 curves in (a)–(d) using least squares. The fitting process used the region between $n = 10^{10}$ and $n = 10^{14}$. Finally, Fig. 8f gives the values of $|F_n|$ for *n* between $10^{10}$ and $10^{18}$ computed using algorithm E.

The experiments confirm the theoretical time complexities of the algorithms. For each of the three algorithms and for each of the two implementations, the slopes are close to the theoretical predictions, i.e., 0.75 for algorithm C and $\frac {2}{3} \approx 0.66$ for algorithms D and E. The slope for algorithm C agrees with the theory up to the third digit after the decimal point. The slopes for the other two algorithms are close to the theoretical predictions, but slightly higher by about 0.01–0.02. In other words, as *n* increases, these algorithms slow down slightly more than the theory predicts.

Even though this difference is small, we investigated it further by measuring the number of CPU instructions in addition to the run time. As described in Supplementary Section S15, that metric agrees with the theory very well. The slight deviation of the run time from the theory is due to practical aspects of code execution, including cache misses and inaccurate branch predictions. On average, this leads to slower execution of CPU instructions. The number of executed instructions, however, agrees with the theory as confirmed by Supplementary Fig. S4. The reason for this is that for our algorithms the total number of executed instructions is not affected by the need to access the main memory more frequently due to more cache misses. This number is also not affected by the decreased efficiency of the instruction pipeline that results from more frequent branch mispredictions.

For memory usage, the theoretically predicted slope is equal to 0.5 for algorithms C and E and $\frac {2}{3}$ for algorithm D. Figure 8 shows that there is a good match between the empirical results and the theoretical predictions for these algorithms. The slopes deviate from the theoretical predictions by less than 0.01 and there does not appear to be a significant positive or negative bias.

The slight jump in time and memory usage of the C version of algorithm E at $n = 10^3$ is due to the implementation of the sieve of Atkin. For small *n* it is optimized to return values from a hard-coded list of small prime numbers. That is, in this case there is no sieving. In our experiments, more computationally intensive sieving starts only when *n* reaches $10^3$. For the Python version, the jump in run time occurs earlier, i.e., between $10^1$ and $10^2$. The reason for this is that our Python code runs the sieve of Atkin in a separate process. This process is started only when $n \ge 32 \approx 10^{1.5}$, which leads to a run time spike at that point. For smaller *n*, algorithm E does not call the sieve of Atkin and the Python code does not start the process.

## Conclusion

This paper introduced several novel formulas for the length $|F_n|$ of a Farey sequence of order *n*. They extend two classic results and combine them in different ways to achieve various trade-offs between iteration and recurrence. The paper also studied the problem of how to efficiently compute $|F_n|$. It described several algorithms that implement the formulas in ways that reduce both the computational time and the memory usage requirements. Our most efficient algorithm runs in $O(n^{2/3})$ time and uses only $O(\sqrt{n})$ memory. These properties make it the most efficient algorithm for computing $|F_n|$ that has been described so far.

Algorithm E is based on formula (12). Even though this formula is long, it leads to the fastest algorithm. It combines the computational optimizations and approaches used by the other algorithms described in the paper. More specifically, it uses the linear sieve to help compute the lengths of Farey sequences of orders up to $\lfloor \sqrt{n} \rfloor$. Next, it enumerates smooth and non-smooth numbers in the interval $[ \alpha , \beta ]$, where $\alpha = \lfloor \sqrt{n} \rfloor + 1$, $\beta = \lfloor n / (v(n) + 1) \rfloor$, and $v(n) = \lfloor n / ( \lfloor \root 3 \of {n^2} \rfloor + 1 ) \rfloor$. The sieve of Atkin is used to enumerate the non-smooth numbers separately from the smooth numbers in order to improve the run time and memory usage while computing the values of $|F_m|$ in that interval. In its final stage, algorithm E uses formula (5) several times to compute $|F_n|$ using previously computed values of $|F_m|$ for $m < n$.

This paper also showed that the empirical time and memory usage of the algorithms agree with the corresponding theoretical time and space computational complexities. The experiments also showed that with algorithm E it is possible to compute the length of the Farey sequence of order $10^{18}$. In other words, this paper makes it possible to explore the properties of $|F_n|$ for larger *n* than was previously possible, given the same amount of computational resources.

Future work could explore the applicability of other prime sieves^29,30^, some of which may be faster and more compact than the sieves used in our algorithms. However, the time and space complexities of our most efficient algorithm are not tied directly to the prime sieves. They result from a combination of theoretical insights and computational techniques. In other words, merely switching to a more efficient prime sieve may not result in better time or space complexity without other changes.

## Methods

The experiments evaluated the run time and memory usage of algorithms C, D, and E (i.e., Algorithms 5, 7, and 8). This was done for two different implementations of the algorithms and their dependencies: one written in C and another in Python. We used version 4.4.7 of GCC, the default C compiler on the experimental platform, to build the C code. It generated native binaries using the 64-bit version of the x86 instruction set. The performance of our Python code was measured with version 3.9.0 of the CPython interpreter^31^, which serves as the reference implementation of the Python language.

### Run time measurements

We used the same high-level Python script to run the evaluation and collect the time and memory usage measurements in all experiments. Each instance of each algorithm ran in a separate process that the evaluation script spawned before running the algorithm. This process exited after completing the run and transmitting the time and memory measurements to the evaluation script. For the Python implementation of the algorithms, this process called the corresponding Python function directly. For the C implementation, we compiled the C code into a shared library from which the evaluation process invoked the corresponding function using Python’s ‘ctypes’ module.

In all cases, the run time of an algorithm was measured using the function ‘time.process_time’ in Python 3, i.e., by subtracting the return value of this function recorded right before launching the algorithm from its value returned right after the algorithm’s completion. We disabled Python’s garbage collector by calling ‘gc.disable()’ before starting an evaluation run. In other words, the run time measurements don’t include the time that Python would normally spend on garbage collection, because the garbage collector was disabled.

### Memory usage measurements

The memory usage of an algorithm was measured by subtracting the peak amount of physical memory used by the spawned process (after initialization but before launching the algorithm) from the peak amount of physical memory recorded after its completion. The memory usage plots report the value of this difference in kilobytes. The script obtained these values from the ‘VmHWM’ record in the special file ‘/proc/[pid]/status’ provided^32^ by our GNU/Linux system, where ‘[pid]’ is the process identifier.

The evaluation script also monitored the number of virtual memory pages transferred from the physical RAM to the designated swap storage on the computer. We used the ‘pswpout’ record in the file ‘/proc/vmstat’ made available by the OS. During the experiments, its value remained the same before and after running each instance of each algorithm, which implies that all virtual memory pages that our program used during these time intervals remained resident in RAM.

### Counting CPU instructions

We used the perf-stat utility^33^ provided by the OS to count the number of central processing unit (CPU) instructions that the code executed. Each process spawned by the evaluation script launched the command ‘perf stat -e instructions -p [pid]’, where ‘[pid]’ was its process identifier. The standard output of perf-stat was redirected to a temporary file. The monitoring process started after initialization but before running the designated algorithm. After the algorithm finished, the spawned process sent the SIGINT signal^34^ to the perf-stat process, which wrote the measurements to its standard output before exiting. The printed text included the number of instructions executed by the spawned process while perf-stat was monitoring. This information, together with the run time and memory usage statistics, was stored for subsequent analysis.

### Averaging results from multiple runs

The logarithms of the run time, the memory usage, and the number of instructions were averaged over 10 independent runs. This was done for each of the two implementations (i.e., C and Python) of the three algorithms (i.e., C, D, and E). The order of the 29 values of *n* between $10^0$ and $10^{14}$ used for the plots was randomized independently in each run. Multiple instances of the algorithms ran in parallel on our server to the extent that they could fit in the available memory without swapping. The number of simultaneously running algorithm instances never exceeded 15. The number of cores on the machine was 32. In other words, there were at least 2 cores available for each algorithm at run time.

### Experimental platform

All results were computed on a 32-core Dell PowerEdge R720 server with 315 gigabytes of RAM. The processor on this machine was 2.20 GHz Intel Xeon E5-2660. The operating system was Red Hat Enterprise Linux (RHEL) version 6.10.

### Native and long integers in Python

Python uses a unified implementation of integer arithmetic that automatically switches from native to long integers when necessary to avoid overflow^35^. On the experimental platform, the size of a native integer was equal to 64 bits. In other words, the Python interpreter automatically switched to long integers whenever it encountered an integer less than $-2^{63}$ or greater than $2^{63} - 1$. This switch occurs for $n > 10^{9.5}$.

### 128-bit integers in C

Our C code used 128-bit integers for all integer values that would not fit in 64 bits for large *n*. More specifically, we built the C code using GCC 4.4.7 and used the ‘__uint128_t’ unsigned 128-bit integer type provided by this compiler.

### Computational model

The theoretical model used for estimating the computational complexity of the algorithms assumes that adding, subtracting, multiplying, dividing, and storing any integer requires *O*(1) time. Similarly, the model assumes that the size of an integer is also in *O*(1). The 128-bit integers used in the C code were sufficiently large to avoid overflow in all experiments. The Python interpreter automatically switched to larger integers when necessary^35^.

### The sieve of Atkin

The experiments used a C implementation of the sieve of Atkin from the ‘primegen’ package^36^, version 0.97, which supports generating prime numbers in any interval $[\alpha , \beta ]$ where $\beta \le 10^{15}$. This bound was sufficiently large for each of our experiments. This implementation uses a relatively small fixed-size static memory buffer instead of dynamic memory allocation. That is, even though in theory the sieve of Atkin requires $O(N^{1/2 + o(1)})$ memory, in practice the memory usage of this particular implementation was constant. For $n > 10^{10}$ the memory usage for computing $|F_n|$ was dominated by the arrays used by our algorithms, i.e., the memory used by the sieve was only a tiny fraction of all memory used by the code.

## Supplementary Information

Supplementary Information.

Supplementary Source Code.

## Data Availability

All data and procedures are described in the main paper or in the supplementary information.

## References

[1] Farey J (1816). On a curious property of vulgar fractions. Philos. Mag..

[2] Hardy G, Wright E (1975). An Introduction to the Theory of Numbers.

[3] Borwein P, Choi S, Rooney B, Weirathmueller A (2007). The Riemann Hypothesis: A Resource for the Afficionado and Virtuoso Alike.

[4] Graham R, Knuth D, Patashnik O (1994). Concrete Mathematics.

[5] Sukhoy V, Stoytchev A (2020). Numerical error analysis of the ICZT algorithm for chirp contours on the unit circle. Sci. Rep..

[6] Sukhoy V, Stoytchev A (2019). Generalizing the inverse FFT off the unit circle. Sci. Rep..

[7] Conway J, Guy R (1995). The Book of Numbers.

[8] Flegg G, Hay C, Moss B (1984). Nicolas Chuquet, Renaissance Mathematician: A Study with Extensive Translation of Chuquet’s Mathematical Manuscript Completed in 1484.

[9] Guthery S (2011). A Motif of Mathematics.

[10] Cauchy A-L (1816). Démonstation d’un theórème curieux sur les nombres (in French). Bulletin des Sciences par la Socièté Philomatique de Paris.

[11] Routledge N (2008). Computing Farey series. Math. Gaz..

[12] Routledge N (2008). Summing Euler’s $$\varphi$$-function. Math. Gaz..

[13] OEIS Foundation Inc. The On-Line Encyclopedia of Integer Sequences. Sequence A005728 (2019). https://oeis.org/A005728. Accessed 23 April 2021.

[14] Gauss, C. F. *Disquisitiones Arithmeticae, Article 39* (Translated by: Clarke, A. Yale University Press, 1965).

[15] Michie D (1968). “Memo” functions and machine learning. Nature.

[16] Crandall R, Pomerance C (2005). Prime Numbers: A Computational Perspective, Chapter 3.

[17] Bach E, Shallit J (1996). Algorithmic Number Theory, Volume 1: Efficient Algorithms.

[18] Gries D, Misra J (1978). A linear sieve algorithm for finding prime numbers. Commun. ACM.

[19] Atkin AOL, Bernstein DJ (2003). Prime sieves using binary quadratic forms. Math. Comput..

[20] Galbraith S (2012). Mathematics of Public Key Cryptography.

[21] Granville A, Buhler J, Stevenhagen P (2008). Smooth numbers: computational number theory and beyond. Algorithmic Number Theory: Lattices, Number Fields, Curves, and Cryptography.

[22] Pomerance, C. The role of smooth numbers in number theoretic algorithms. In *Proceedings of the International Congress of Mathematicians*, 411–422 (Birkhäuser, 1995).

[23] Pătraşcu, C. & Pătraşcu, M. Computing order statistics in the Farey sequence. In *Proceedings of the 6th International Symposium on Algorithmic Number Theory* (ed. Buell, D.) 358–366 (Burlington, 2004).

[24] Pawlewicz, J. Order statistics in the Farey sequences in sublinear time. In *Proceedings of the European Symposium on Algorithms*, 218–229 (Eilat, 2007).

[25] Pawlewicz J, Pătraşcu M (2009). Order statistics in the Farey sequences in sublinear time and counting primitive lattice points in polygons. Algorithmica.

[26] Deléglise M, Rivat J (1996). Computing the summation of the Möbius function. Exp. Math..

[27] Hurst G (2018). Computations of the Mertens function and improved bounds on the Mertens conjecture. Math. Comput..

[28] Ye Y (1994). Combining binary search and Newton’s method to compute real roots for a class of real functions. J. Complex..

[29] Pritchard P (1981). A sublinear additive sieve for finding prime numbers. Commun. ACM.

[30] Sorenson J (2015). Two compact incremental prime sieves. LMS J. Comput. Math..

[31] The Python Software Foundation. Python Release 3.9.0 (2020). https://www.python.org/downloads/release/python-390/. Accessed 23 April 2021.

[32] The Linux man-pages project. proc—process information pseudo-filesystem (2020). https://man7.org/linux/man-pages/man5/proc.5.html. Accessed 23 April 2021.

[33] The Linux man-pages project. perf-stat—Run a command and gather performance counter statistics (2020). https://man7.org/linux/man-pages/man1/perf-stat.1.html. Accessed 23 April 2021.

[34] The Linux man-pages project. signal—overview of signals (2020). https://man7.org/linux/man-pages/man7/signal.7.html. Accessed 23 April 2021.

[35] Zadka, M. & van Rossum, G. PEP 237—Unifying Long Integers and Integers (2001). https://www.python.org/dev/peps/pep-0237/. Accessed 23 April 2021.

[36] Bernstein, D. J. Primegen: A small, fast library for generating prime numbers in order. (1999). Version 0.97. https://cr.yp.to/primegen.html. Accessed 23 April 2021.

