# Supplementary material for: Formulas and algorithms for the length of a Farey sequence
Source: Sci Rep. 2021 Nov 15;11:22218. doi: 10.1038/s41598-021-99545-w (PMC8593030; doi:10.1038/s41598-021-99545-w)
Supplement: Supplementary file 1 — Supplementary Information. [file 41598_2021_99545_MOESM1_ESM.pdf]

Supplementary Information for "Formulas and Algorithms for the Length of a Farey Sequence"

Vladimir Sukhoy[1] & Alexander Stoytchev[1,*]

[1] Department of Electrical and Computer Engineering, Iowa State University, Ames, IA 50011, USA. Correspondence and requests for materials should be addressed to A.S. (email: alexs@iastate.edu).

S1. Algorithm for Enumerating and Counting the Elements of a Farey Sequence

For the Farey sequence F_n, the mediant property implies that the numerator p and the denominator q of the *third* fraction in a triple of adjacent fractions $({}^a/_b, {}^c/_d, {}^p/_q)$ can be expressed in terms of the values of a, b, c, and d as follows:

$$p = \left\lfloor \frac{n+b}{d} \right\rfloor c - a, \quad \text{and} \quad q = \left\lfloor \frac{n+b}{d} \right\rfloor d - b. \tag{15}$$

This pair of formulas enables an elegant algorithm[11] for enumerating the elements of F_n. The main idea is to use a window of adjacent fractions $({}^a/_b, {}^c/_d)$ that 'slides' over the elements of F_n until it reaches ${}^1/_1$. This window is initialized with the pair of fractions $({}^0/_1, {}^1/_n)$ that are the first two elements of the sequence F_n.

Algorithm S1 uses this approach to compute the length of the Farey sequence of order n by explicitly enumerating its elements and counting them. Because the length of F_n grows quadratically with n, this algorithm runs in $O(n^2)$ time, which makes it too slow for large values of n. Nevertheless, for small n, it can be used to cross-check the results of the faster algorithms described in the main paper. This algorithm uses a tiny amount of memory because it only prints the sequence elements and does not need to store them in memory. In other words, its space complexity is $O(1)$.

Algorithm S1. Enumerate all elements of the Farey sequence F_n and return $|F_n|$. Runs in $O(n^2)$ time and uses $O(1)$ memory.

```
function EnumerateFareyFractions(n)
    s ← 0;
    (a, b, c, d) ← (0, 1, 1, n);
    while true do
        s ← s + 1;
        Print(a, " / ", b);
        if a = b then
            break;
        end if
        k ← ⌊(n+b)/d⌋;
        (a, b, c, d) ← (c, d, kc − a, kd − b);
    end while
    return s;
end function
```

S2. Summary of the Time and Memory Complexities of the Algorithms

The main paper describes five algorithms for computing the length of a Farey sequence. To distinguish between them, the algorithms are denoted with the first five letters of the alphabet. These letters are also used as suffixes in the function name for each algorithm, e.g., FareyLengthA or FareyLengthE.

The algorithms are presented from the slowest to the fastest. This is done in order to explain the computational techniques and optimizations that were needed to derive the most efficient algorithm, i.e., algorithm E, which runs in $O(n^{2/3})$ time and uses $O(\sqrt{n})$ memory. Figure S1 provides a visual overview of the time and memory complexities of all five algorithms.

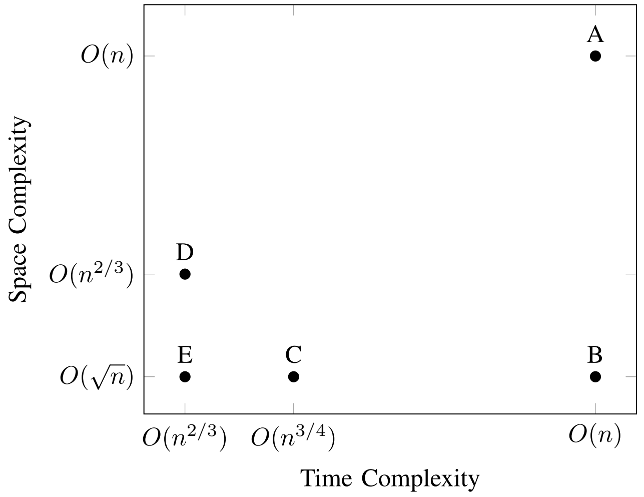

Figure S1: Visual summary of the time and space complexities of the five algorithms for computing the length of the Farey sequence of order n that are described in the main paper.

S3. COMPUTING EULER'S TOTIENT FUNCTION

This section defines Euler's totient function $\varphi(n)$ and proves some of its properties. It also gives the pseudo-code for two algorithms that can be used to compute it. An example that illustrates these algorithms is also provided.

Definition 1. *Euler's totient function $\varphi(n)$ maps each positive integer n to the number of integers in the range $[1, n]$ that are coprime with n. More formally,*

$$\varphi(n) = \left|\left\{k \in \{1, 2, \ldots, n\} \text{ s.t. } \gcd(n, k) = 1\right\}\right|. \tag{16}$$

Theorem 1. *For each $n > 1$, the length of the Farey sequence of order n can be expressed as the sum of $\varphi(n)$ and the length of the Farey sequence of order $n - 1$. That is,*

$$|F_n| = |F_{n-1}| + \varphi(n), \qquad \text{for each } n > 1. \tag{17}$$

Proof. Let D_n be a set that contains the elements of the sequence F_n that are not members of the sequence F_{n-1}, i.e.,

$$D_n = \left\{{}^{p}/_{q} \in F_n \ \text{ s.t. } \ {}^{p}/_{q} \notin F_{n-1}\right\}. \tag{18}$$

Then,

$$|F_n| = |F_{n-1}| + |D_n|. \tag{19}$$

It remains to show that $|D_n| = \varphi(n)$. For each irreducible fraction ${}^{p}/_{q} \in D_n$ the denominator q must be equal to n, otherwise ${}^{p}/_{q}$ would be in F_{n-1}. Thus, the cardinality of D_n is equal to the number of positive integers p between 1 and n for which the fraction ${}^{p}/_{n}$ is irreducible. This fraction is irreducible if and only if p is coprime with n. Therefore, $|D_n| = \varphi(n)$, which completes the proof. □

Theorem 2. *For each positive integer $n \geq 1$ the length of the Farey sequence of order n can be computed by adding 1 to the sum of the values of Euler's totient function from 1 to n, i.e.,*

$$|F_n| = 1 + \sum_{k=1}^{n} \varphi(k). \tag{20}$$

Proof. For $n = 1$ the formula reduces to $|F_1| = 1 + \varphi(1) = 1 + 1 = 2$, which is correct. For $n > 1$ the proof follows from Theorem 1, i.e.,

$$\begin{aligned}
|F_n| &= |F_{n-1}| + \varphi(n) \\
&= |F_{n-2}| + \varphi(n-1) + \varphi(n) \\
&= |F_{n-3}| + \varphi(n-2) + \varphi(n-1) + \varphi(n) \\
&\ldots \\
&= |F_1| + \varphi(2) + \varphi(3) + \cdots + \varphi(n) \\
&= 1 + \varphi(1) + \varphi(2) + \varphi(3) + \cdots + \varphi(n) \\
&= 1 + \sum_{k=1}^{n} \varphi(k).
\end{aligned} \tag{21}$$

□

Table S1 shows the values of Euler's totient function $\varphi(k)$ for all integer arguments k from 1 and 20. Table S2 shows the length of the Farey sequence F_n for values of n from 1 to 20. Using these numbers we can verify that formula (20) holds for some small values of n. For example, $|F_5| = 1 + \varphi(1) + \varphi(2) + \varphi(3) + \varphi(4) + \varphi(5) = 11$.

k	1	2	3	4	5	6	7	8	9	10	11	12	13	14	15	16	17	18	19	20
$\varphi(k)$	1	1	2	2	4	2	6	4	6	4	10	4	12	6	8	8	16	6	18	8

Table S1: Values of Euler's totient function $\varphi(k)$ for k between 1 and 20.

n	1	2	3	4	5	6	7	8	9	10	11	12	13	14	15	16	17	18	19	20
$\|F_n\|$	2	3	5	7	11	13	19	23	29	33	43	47	59	65	73	81	97	103	121	129

Table S2: The length of the Farey sequence $|F_n|$ for n between 1 and 20.

The following theorem gives a formula for computing the value of $\varphi(p \cdot k)$, where p is a prime number and k is any positive integer. This formula is used as an update rule by several algorithms described in this manuscript.

Theorem 3. *Let p be a prime number and let k be a positive integer. Then, the value of Euler's totient $\varphi(p \cdot k)$ can be expressed as follows:*

$$\varphi(p \cdot k) = \begin{cases} p \cdot \varphi(k), & \text{if } p \text{ is a factor of } k, \\ (p-1) \cdot \varphi(k), & \text{if } p \text{ is not a factor of } k. \end{cases} \tag{22}$$

Proof. Euler's product formula allows us to express the values of $\varphi(k)$ and $\varphi(p \cdot k)$ as shown below:

$$\varphi(k) = k \prod_{q|k} \left(1 - \frac{1}{q}\right), \tag{23}$$

$$\varphi(p \cdot k) = p \cdot k \prod_{q|k \cdot p} \left(1 - \frac{1}{q}\right), \tag{24}$$

where the products run over the distinct prime numbers q that divide k and $k \cdot p$, respectively.

If p is a factor of k, then $\varphi(p \cdot k)$ can be expressed as follows:

$$\varphi(p \cdot k) = p \cdot k \prod_{q|k \cdot p} \left(1 - \frac{1}{q}\right) = p \cdot k \prod_{q|k} \left(1 - \frac{1}{q}\right) = p \cdot \varphi(k), \tag{25}$$

which proves the first branch in formula (22).

If p is not a factor of k, then formulas (23) and (24) imply that $\varphi(p \cdot k)$ has the following value:

$$\varphi(p \cdot k) = p \cdot k \prod_{q|k \cdot p} \left(1 - \frac{1}{q}\right) = p \cdot k \prod_{q|k} \left(1 - \frac{1}{q}\right)\left(1 - \frac{1}{p}\right) = p\left(1 - \frac{1}{p}\right) k \prod_{q|k} \left(1 - \frac{1}{q}\right) = (p-1) \cdot \varphi(k), \tag{26}$$

which proves the second branch in formula (22). □

One way to compute Euler's totients for all integers between 1 and N is to use formula (22) with the array L_p that the linear sieve algorithm[18] generates. Algorithm S2 gives the pseudo-code for the linear sieve. Its main goal is to compute a list P that consists of all prime numbers in the interval $[1, N]$. The algorithm solves this problem in $O(N)$ time and uses $O(N)$ memory. In addition to P, the linear sieve algorithm also fills an array L_p that contains the smallest prime factor for each integer in the interval $[1, N]$ (the algorithm sets the value of $L_p[1]$ to 1 as a special case). The array L_p is sufficient for computing the Euler's totients $\varphi(1), \varphi(2), \ldots, \varphi(N)$ using a special case of formula (22) as described below.

Algorithm S3 fills the array φ such that its elements $\varphi[1], \varphi[2], \ldots, \varphi[N]$ are equal to Euler's totients $\varphi(1), \varphi(2), \ldots, \varphi(N)$. The algorithm starts by setting $\varphi[1]$ to $\varphi(1)$, which is equal to 1. Then, the algorithm iterates over m from 2 to N and sets $\varphi[m]$ to Euler's totient $\varphi(m)$. Each iteration of this for-loop uses the following version of formula (22):

$$\varphi(m) = \begin{cases} p \cdot \varphi(k), & \text{if } L_p[k] = p, \\ (p-1) \cdot \varphi(k), & \text{if } L_p[k] \neq p, \end{cases} \tag{27}$$

where $p = L_p[m]$ is the smallest prime number that divides m and $k = \lfloor m/p \rfloor = m/p$ (we use the floor function to indicate that integer division should be used here). In other words, $m = k \cdot p$. Because p is the smallest prime number that divides m, the prime factorization of m can be derived from the prime factorization of k by adding one more factor p to it. This also implies that the smallest prime factor of k must be greater than or equal to p, i.e., $L_p[k] \geq L_p[k \cdot p] = p$. Moreover, p is a factor of k if and only if $L_p[k] = p$. Conversely, if $L_p[k] \neq p$, then the prime number p is not a factor of k. This logic leads to formula (27). Algorithm S3 runs in $O(N)$ time because it performs $O(1)$ operations for each integer between 1 and N. The space complexity of this algorithm is also in $O(N)$ because it creates an array φ that consists of N integers.

Algorithm S2. Find all prime numbers in the interval $[2, N]$ using a linear sieve. This algorithm also returns an array L_p that contains the smallest prime factor for each integer in $[2, N]$. Runs in $O(N)$ time and uses $O(N)$ memory.

```
function LinearSieve(N)
    P ← EmptyList( );                // list of all prime numbers in the interval [2, N]
    L_p ← NullArray(N+1);
    L_p[1] ← 1;                      // array of smallest prime factors
    for k ← 2 to N do
        if L_p[k] is null then
            // k is a prime number
            L_p[k] ← k;
            Append(P, k);
        end if
        for p in P do
            q ← p · k;
            if p > L_p[k] or q > N then
                break;
            end if
            L_p[q] ← p;
        end for
    end for
    return (P, L_p);
end function
```

Algorithm S3. Compute Euler's totient function $\varphi(m)$ for each $m \in \{1, 2, \ldots, N\}$ by traversing the array of smallest prime factors L_p generated by the linear sieve algorithm. This algorithm runs in $O(N)$ time and uses $O(N)$ memory.

```
function ComputeTotients(N, L_p)
    φ ← EmptyArray(N+1);             // values of Euler's totient
    φ[1] ← 1;
    for m ← 2 to N do
        p ← L_p[m];
        k ← ⌊m/p⌋;
        if L_p[k] = p then
            φ[m] ← p · φ[k];
        else
            φ[m] ← (p − 1) · φ[k];
        end if
    end for
    return φ;
end function
```

Figure S2 visualizes a sample run of the linear sieve (i.e., Algorithm S2) with $N = 5$. It shows the state of the array of smallest prime factors L_p and the list of prime numbers P for $k \in \{1, 2, 3, 4, 5\}$. The elements of L_p and P that change after each iteration are colored in red. All elements of L_p are initially set to null, which is indicated with $\emptyset$ in the figure. The list P starts empty.

k	L_p	P
1	[1, ∅, ∅, ∅, ∅]	()
2	[1, 2, ∅, 2, ∅]	(2)
3	[1, 2, 3, 2, ∅]	(2, 3)
4	[1, 2, 3, 2, ∅]	(2, 3)
5	[1, 2, 3, 2, 5]	(2, 3, 5)

Figure S2: Illustration of the linear sieve algorithm for $N = 5$.

The algorithm starts by setting $L_p[1]$ to 1. Then, it proceeds to the for-loop that starts at $k = 2$. Because $L_p[k]$ is null at the beginning of this iteration, the algorithm marks $k = 2$ as a prime number by setting $L_p[2]$ to 2 and appending 2 to the list P. Subsequently, the algorithm iterates over each p in $P = (2)$, sets the variable q to the product $p \cdot k$, and then sets $L_p[q]$ to p until q exceeds N. In our example, this leads to setting $L_p[4]$ to 2. Next, the algorithm proceeds to $k = 3$. Because $L_p[k]$ is null here, this value of k is also a prime number, which leads to setting $L_p[3]$ to 3 and appending 3 to P. No other elements of L_p change because $2 \cdot 3 > N = 5$, which leads to breaking out of the inner for-loop on its first iteration. Subsequently, the algorithm continues to $k = 4$. Because $L_p[k]$ is not null, this value of k is not a prime number and the list P remains unchanged here. The inner for-loop also terminates early and does not modify any elements of L_p. Finally, the algorithm advances to $k = 5$. The value of $L_p[k]$ is null here, which leads to setting $L_p[5]$ to 5 and appending 5 to the list P (i.e., 5 is a prime number). The inner for loop, again, terminates early and the function returns the final state of P and L_p.

Figure S3 shows an example for Algorithm S3 with $N = 5$. This algorithm computes Euler's totients $\varphi(1)$, $\varphi(2)$, $\varphi(3)$, $\varphi(4)$, and $\varphi(5)$ using the array L_p computed by the linear sieve algorithm in the previous example, i.e., $L_p = [1, 2, 3, 2, 5]$. Algorithm S3 stores the computed totients in the array φ, which starts uninitialized. The uninitialized elements of φ are denoted with the symbol $\emptyset$ in the figure.

m	L_p	$p = L_p[m]$	$k = \lfloor m/p \rfloor$	$L_p[k]$	φ	Computing $\varphi[m]$
1	[1, 2, 3, 2, 5]	1	1	1	[1, ∅, ∅, ∅, ∅]	$\varphi[1] = 1$
2	[1, 2, 3, 2, 5]	2	1	1	[1, 1, ∅, ∅, ∅]	$\varphi[2] = (p-1) \cdot \varphi[k] = (2-1) \cdot 1 = 1$
3	[1, 2, 3, 2, 5]	3	1	1	[1, 1, 2, ∅, ∅]	$\varphi[3] = (p-1) \cdot \varphi[k] = (3-1) \cdot 1 = 2$
4	[1, 2, 3, 2, 5]	2	2	2	[1, 1, 2, 2, ∅]	$\varphi[4] = p \cdot \varphi[k] = 2 \cdot 1 = 2$
5	[1, 2, 3, 2, 5]	5	1	1	[1, 1, 2, 2, 4]	$\varphi[5] = (p-1) \cdot \varphi[k] = (5-1) \cdot 1 = 4$

Figure S3: Illustration of the algorithm for computing Euler's totients with $N = 5$.

The algorithm starts by setting $\varphi[1]$ to 1. Then, it continues to the for-loop, which starts at $m = 2$. It sets the value of the variable p to $L_p[m]$, which in this case is equal to 2. The variable k is set to $\lfloor m/p \rfloor = \lfloor 2/2 \rfloor = 1$. In this iteration, $L_p[k] \neq p$ and therefore the value of $\varphi[m]$ is set to $(p-1) \cdot \varphi[k] = (2-1) \cdot \varphi[1] = 1$. Next, the algorithm proceeds to $m = 3$. It sets the variable p to $L_p[m]$, which is equal to 3 and sets the variable k to $\lfloor m/p \rfloor$, which is equal to 1. The if-statement, again, sets the value of $\varphi[m]$ to $(p-1) \cdot \varphi[k] = (3-1) \cdot 1 = 2$. Subsequently, the algorithm proceeds to $m = 4$. In this iteration, p becomes 2 because $L_p[4] = 2$. The variable k is also set to 2, because $\lfloor m/p \rfloor = \lfloor 4/2 \rfloor = 2$. In this case, $L_p[k] = p$ and therefore the first branch of the if-statement is executed, which sets $\varphi[m]$ to $p \cdot \varphi[k] = 2 \cdot \varphi[2] = 2$. Finally, the algorithm continues to $m = 5$. Here, p becomes equal to $5 = L_p[m]$ and k becomes equal to $1 = \lfloor 5/5 \rfloor$. Because $L_p[k] \neq p$, the value of $\varphi[m]$ is set to $(p-1) \cdot \varphi[k] = (5-1) \cdot 1 = 4$. After the last iteration, the function returns the final state of the array φ.

S4. Lemmas Used in the Proofs

The following lemma is used in the proof of Theorem 13.

Lemma 4. *Let k and n be two positive integers and let $S(k, n)$ be a set of all integers m such that $\lfloor n/m \rfloor = k$, i.e.,*

$$S(k, n) = \big\{ m \in \mathbb{N} \ \ s.t. \ \ \lfloor n/m \rfloor = k \big\}. \tag{28}$$

Then, the set $S(k, n)$ consists of all integers in the interval $\big(\lfloor \frac{n}{k+1} \rfloor, \lfloor \frac{n}{k} \rfloor\big]$, i.e.,

$$S(k, n) = \left\{ \left\lfloor \frac{n}{k+1} \right\rfloor + 1, \left\lfloor \frac{n}{k+1} \right\rfloor + 2, \ldots, \left\lfloor \frac{n}{k} \right\rfloor \right\}. \tag{29}$$

Moreover, if $k_1 \neq k_2$, then the sets $S(k_1, n)$ and $S(k_2, n)$ are disjoint, i.e.,

$$S(k_1, n) \cap S(k_2, n) = \emptyset, \quad \text{if } \ k_1 \neq k_2. \tag{30}$$

Finally, the union of the sets $S(k, n)$ over all $k \in \{1, 2, \ldots, \lfloor \sqrt{n} \rfloor\}$ is equal to the set of integers that fall in the interval $[u(n)+1, n]$, where $u(n) = \left\lfloor \frac{n}{\lfloor \sqrt{n} \rfloor + 1} \right\rfloor$. That is,

$$\bigcup_{k=1}^{\lfloor \sqrt{n} \rfloor} S(k, n) = \{u(n) + 1, \, u(n) + 2, \ldots, \, n\}. \tag{31}$$

Proof. First, we will prove formula (29). Let $m \in S(k, n)$. Then, $\lfloor n/m \rfloor = k$, i.e.,

$$k \leq \frac{n}{m} < k + 1. \tag{32}$$

Reciprocating the three terms leads to:

$$\frac{1}{k+1} < \frac{m}{n} \leq \frac{1}{k}. \tag{33}$$

Multiplying this inequality by n leads to the following interval for the value of m:

$$\frac{n}{k+1} < m \leq \frac{n}{k}. \tag{34}$$

Because m is an integer, the floor function can be applied to the lower and upper bounds for the value of m in the previous inequality, i.e.,

$$\left\lfloor \frac{n}{k+1} \right\rfloor < m \leq \left\lfloor \frac{n}{k} \right\rfloor. \tag{35}$$

Therefore, $m \in \big(\lfloor \frac{n}{k+1} \rfloor, \lfloor \frac{n}{k} \rfloor\big]$, which implies that $S(k, n) \subseteq \big(\lfloor \frac{n}{k+1} \rfloor, \lfloor \frac{n}{k} \rfloor\big]$.

Conversely, let m be an integer that lies in the interval $\big(\lfloor \frac{n}{k+1} \rfloor, \lfloor \frac{n}{k} \rfloor\big]$. That is,

$$\left\lfloor \frac{n}{k+1} \right\rfloor + 1 \leq m \leq \left\lfloor \frac{n}{k} \right\rfloor. \tag{36}$$

Dividing by n and reciprocating leads to:

$$\frac{n}{\lfloor \frac{n}{k} \rfloor} \leq \frac{n}{m} \leq \frac{n}{\lfloor \frac{n}{k+1} \rfloor + 1}. \tag{37}$$

Because $\lfloor n/k \rfloor \leq n/k$, the lower bound can be stated as:

$$k = \frac{n}{\frac{n}{k}} \leq \frac{n}{\lfloor \frac{n}{k} \rfloor} \leq \frac{n}{m}. \tag{38}$$

On the other hand, $\lfloor \frac{n}{k+1} \rfloor + 1 > \frac{n}{k+1}$. Therefore, the upper bound in (37) can be expressed as:

$$\frac{n}{m} \leq \frac{n}{\lfloor \frac{n}{k+1} \rfloor + 1} < \frac{n}{\frac{n}{k+1}} = k + 1. \tag{39}$$

Inequalities (38) and (39) imply that $k \leq n/m < k + 1$ and, thus, $\lfloor n/m \rfloor = k$. Therefore, $\mathbb{N} \cap \big(\lfloor \frac{n}{k+1} \rfloor, \lfloor \frac{n}{k} \rfloor\big] \subseteq S(k, n)$, which proves formula (29).

Formula (30) is proven by contradiction. Suppose that there is a pair of integers k_1 and k_2 such that $k_1 \neq k_2$ and that the intersection of $S(k_1, n)$ and $S(k_2, n)$ is not empty. Then, there is an integer m that is an element of both sets, which implies that $\lfloor n/m \rfloor = k_1$ and $\lfloor n/m \rfloor = k_2$. Thus, $k_1 = k_2$, which is a contradiction that proves formula (30).

Formula (31) is proven by showing that the union of the sets in the left-hand side forms a contiguous range of integers that matches the set in the right-hand side. In this case, larger values of k correspond to smaller elements of $S(k,n)$. From formula (29) we can derive that the maximum element in the set $S(k+1,n)$ can be obtained by decrementing the minimum element in the set $S(k,n)$. More formally,

$$\max S(k+1,n) = \left\lfloor \frac{n}{k+1} \right\rfloor = \left\lfloor \frac{n}{k+1} \right\rfloor + 1 - 1 = \min S(k,n) - 1. \tag{40}$$

The minimum element in the set $S(\lfloor\sqrt{n}\rfloor, n)$ is equal to $\left\lfloor \frac{n}{\lfloor\sqrt{n}\rfloor+1} \right\rfloor + 1 = u(n) + 1$. The maximum element in the set $S(1,n)$ is equal to n. Thus, all integers between $u(n)+1$ and n are elements of the union. □

Many formulas in this manuscript use the function $u(n) = \lfloor n/(\lfloor\sqrt{n}\rfloor + 1)\rfloor$ to simplify the notation. Depending on the value of the integer n, this function evaluates to either $\lfloor\sqrt{n}\rfloor - 1$ or $\lfloor\sqrt{n}\rfloor$. For example, if $n = 4$, then it evaluates to $1 = \lfloor\sqrt{4}\rfloor - 1$. For $n = 6$, however, it evaluates to $2 = \lfloor\sqrt{6}\rfloor$. The next lemma formally proves this property.

Lemma 5. *For any positive integer n, the value of the function $u(n) = \lfloor {}^{n}/(\lfloor\sqrt{n}\rfloor+1) \rfloor$ is equal to either $\lfloor\sqrt{n}\rfloor - 1$ or $\lfloor\sqrt{n}\rfloor$.*

Proof. For the fraction in the definition of $u(n)$, the value of the denominator lies between $\sqrt{n}$ and $\sqrt{n}+1$, i.e.,

$$\sqrt{n} < \lfloor\sqrt{n}\rfloor + 1 \leq \sqrt{n} + 1. \tag{41}$$

Reciprocating all three values changes the direction of the inequality and leads to:

$$\frac{1}{\sqrt{n}+1} \leq \frac{1}{\lfloor\sqrt{n}\rfloor + 1} < \frac{1}{\sqrt{n}}. \tag{42}$$

Multiplying by n leads to the following bounds for the fraction in $u(n)$ that is used as the argument of the floor function:

$$\frac{n}{\sqrt{n}+1} \leq \frac{n}{\lfloor\sqrt{n}\rfloor + 1} < \frac{n}{\sqrt{n}} = \sqrt{n}. \tag{43}$$

The value of the term $n/(\sqrt{n}+1)$ in the left-hand side can be expressed as follows:

$$\frac{n}{\sqrt{n}+1} = \frac{n-1+1}{\sqrt{n}+1} = \frac{(\sqrt{n}-1)(\sqrt{n}+1)+1}{\sqrt{n}+1} = \sqrt{n} - 1 + \frac{1}{\sqrt{n}+1}. \tag{44}$$

Therefore,

$$\sqrt{n} - 1 < \frac{n}{\sqrt{n}+1}. \tag{45}$$

Combining inequality (43) with inequality (45) leads to the following formula:

$$\sqrt{n} - 1 < \frac{n}{\lfloor\sqrt{n}\rfloor + 1} < \sqrt{n}. \tag{46}$$

Applying the floor function to the previous inequality makes the middle term equal to $u(n)$. It also changes the inequality such that it is no longer strict when $\sqrt{n}$ is an integer. This completes the proof, i.e.,

$$\lfloor\sqrt{n}\rfloor - 1 = \lfloor\sqrt{n} - 1\rfloor \leq u(n) \leq \lfloor\sqrt{n}\rfloor, \tag{47}$$

where $\lfloor\sqrt{n}\rfloor - 1 = \lfloor\sqrt{n} - 1\rfloor$ follows from the definition of the floor function. □

The following two lemmas are used in the proof of Theorem 12. They express the value of $\lfloor\frac{n+1}{k}\rfloor$ in two ways depending on whether k divides $n+1$ or not.

Lemma 6. *Let n be a positive integer and let k be an integer between 1 and n. If k does not divide $n+1$, then the value of $\lfloor\frac{n+1}{k}\rfloor$ is equal to $\lfloor\frac{n}{k}\rfloor$. That is,*

$$\left\lfloor \frac{n+1}{k} \right\rfloor = \left\lfloor \frac{n}{k} \right\rfloor, \quad \textit{if } (n{+}1) \bmod k \neq 0. \tag{48}$$

Proof. The properties of integer division imply that there is exactly one pair of integers x and y such that the value of $n+1$ can be expressed in the following form:

$$n + 1 = xk + y, \tag{49}$$

where $0 \leq y < k$. In this case, $x = \lfloor\frac{n+1}{k}\rfloor$ is the quotient and $y = (n{+}1) \bmod k$ is the remainder. The lemma states that k does not divide $n+1$, which implies that y cannot be zero, i.e., $1 \leq y < k$.

Subtracting 1 from both sides of equation (49) leads to the following expression for the value of n:

$$n = xk + y - 1. \tag{50}$$

The value of the term $y - 1$ is an integer that lies in the interval $[0, k-1)$. Once again, the uniqueness of integer division implies that $y - 1 = n \bmod k$ and that $x = \left\lfloor \frac{n}{k} \right\rfloor$, which completes the proof. □

Lemma 7. *Let n be a positive integer. Also, let k be an integer between 1 and n. If k divides $n+1$, then the value of $\left\lfloor \frac{n+1}{k} \right\rfloor$ is a whole number equal to $\left\lfloor \frac{n}{k} \right\rfloor + 1$, i.e.,*

$$\left\lfloor \frac{n+1}{k} \right\rfloor = \frac{n+1}{k} = \left\lfloor \frac{n}{k} \right\rfloor + 1, \quad \textit{if } (n+1) \bmod k = 0. \tag{51}$$

Proof. Similarly to the previous proof, the properties of integer division imply that:

$$n + 1 = xk + y = xk, \tag{52}$$

where $x = \left\lfloor \frac{n+1}{k} \right\rfloor$ and $y = (n+1) \bmod k = 0$. In this case, $y = 0$ because $n + 1$ is evenly divisible by k.

Once again, we can subtract 1 from both sides of (52), which leads to the following formula for the value of n:

$$n = xk - 1. \tag{53}$$

Furthermore, we can express xk as $(x-1)k + k$, which leads to

$$n = (x-1)k + k - 1. \tag{54}$$

Here the uniqueness of integer division implies that $\left\lfloor \frac{n}{k} \right\rfloor = x - 1$. Recalling that $x = \left\lfloor \frac{n+1}{k} \right\rfloor$ completes the proof. □

The next lemma proves an interesting property of the floor function, which is used in the correctness proof for Algorithm C.

Lemma 8. *Let k, j, and n be three positive integers. Then,*

$$\left\lfloor \frac{\lfloor n/k \rfloor}{j} \right\rfloor = \left\lfloor \frac{n/k}{j} \right\rfloor. \tag{55}$$

Proof. Similarly to the previous two lemmas, we start by using integer division to express the value of $\lfloor n/k \rfloor$ as follows:

$$\lfloor n/k \rfloor = xj + y, \tag{56}$$

where $x = \left\lfloor \frac{\lfloor n/k \rfloor}{j} \right\rfloor$ and $y = \lfloor n/k \rfloor \bmod j$. The integer y lies in the closed interval $[0, j-1]$.

The value of n/k is equal to the sum of its integer and fractional parts, i.e.,

$$n/k = \lfloor n/k \rfloor + f, \tag{57}$$

where $f = n/k - \lfloor n/k \rfloor$. Because n and k are integers, the value of f is an element of the closed interval $\left[0, \frac{k-1}{k}\right]$, i.e., it never reaches 1.

Combining (56) and (57) allows us to derive the following formula for the value of $\frac{n/k}{j}$:

$$\frac{n/k}{j} = \frac{\lfloor n/k \rfloor}{j} + \frac{f}{j} = \frac{xj + y}{j} + \frac{f}{j} = x + \frac{y}{j} + \frac{f}{j}. \tag{58}$$

This formula, in turn, implies that the following upper and lower bounds hold for $\frac{n/k}{j}$:

$$x = \left\lfloor \frac{\lfloor n/k \rfloor}{j} \right\rfloor \leq \frac{\lfloor n/k \rfloor}{j} \leq \frac{n/k}{j} < x + \frac{j-1}{j} + \frac{1}{j} = x + 1. \tag{59}$$

In other words, $x \leq \frac{n/k}{j} < x + 1$. Applying the floor function to all three terms leads to $x \leq \left\lfloor \frac{n/k}{j} \right\rfloor < x + 1$, since x is an integer. Because the term in the middle must be an integer and because the second inequality remains strict, it follows that $\left\lfloor \frac{n/k}{j} \right\rfloor$ must be equal to $x = \left\lfloor \frac{\lfloor n/k \rfloor}{j} \right\rfloor$. This completes the proof. □

Lemma 9. *Let k, j, and n be three positive integers such that $n \geq kj$. Then, the lengths of the following two Farey sequences are equal:*

$$\left| F_{\lfloor \lfloor n/k \rfloor / j \rfloor} \right| = \left| F_{\lfloor n/(kj) \rfloor} \right|. \tag{60}$$

Proof. The proof follows from Lemma 8, which proved that the values of the two subindices in (60) are equal. Also, when $n \geq kj$, the value of $\lfloor n/(kj) \rfloor$ is an integer that is greater than or equal to 1, i.e., it identifies an order of a Farey sequence. □

The next two lemmas are used to prove the computational complexities of FAREYLENGTHL and FAREYLENGTHC (i.e., Algorithm S10 and Algorithm 5), respectively.

Lemma 10. *Let N be a positive integer. Then,*

$$\sum_{k=1}^{N} O\left(\frac{1}{k}\right) = O(\log N). \tag{61}$$

Proof. Let $f(x) = \frac{1}{x}$. This function is monotonically decreasing in the interval $(0, \infty)$. Therefore,

$$\int_{k}^{k+1} \frac{1}{x}\,\mathrm{d}x < \frac{1}{k} < \int_{k-1}^{k} \frac{1}{x}\,\mathrm{d}x, \tag{62}$$

for each $k \in \mathbb{N} = \{1, 2, \dots\}$. This implies that we can find lower and upper bounds for the sum in formula (61) as follows:

$$\int_{1}^{N+1} \frac{1}{x}\,\mathrm{d}x = \sum_{k=1}^{N} \int_{k}^{k+1} \frac{1}{x}\,\mathrm{d}x < \sum_{k=1}^{N} \frac{1}{k} = 1 + \sum_{k=2}^{N} \frac{1}{k} \leq 1 + \sum_{k=2}^{N} \int_{k-1}^{k} \frac{1}{x}\,\mathrm{d}x = 1 + \int_{1}^{N} \frac{1}{x}\,\mathrm{d}x. \tag{63}$$

The left-most integral is equal to:

$$\int_{1}^{N+1} \frac{1}{x}\,\mathrm{d}x = \ln x \,\Big|_{1}^{N+1} = \ln(N+1). \tag{64}$$

The right-most term in this inequality evaluates to:

$$1 + \int_{1}^{N} \frac{1}{x}\,\mathrm{d}x = 1 + \ln x \,\Big|_{1}^{N} = 1 + \ln N. \tag{65}$$

Therefore, the bounds for the sum are:

$$\ln(N+1) < \sum_{k=1}^{N} \frac{1}{k} \leq 1 + \ln N, \tag{66}$$

which completes the proof. □

Lemma 11. *Let N be a positive integer. Then,*

$$\sum_{k=1}^{N} O\left(\frac{1}{\sqrt{k}}\right) = O\left(\sqrt{N}\right). \tag{67}$$

Proof. Let $f(x) = \frac{1}{\sqrt{x}}$. This function is monotonically decreasing in the interval $(0, \infty)$. Thus, for each $k \in \mathbb{N} = \{1, 2, \dots\}$ the following inequality holds:

$$\int_{k}^{k+1} \frac{1}{\sqrt{x}}\,\mathrm{d}x < \frac{1}{\sqrt{k}} < \int_{k-1}^{k} \frac{1}{\sqrt{x}}\,\mathrm{d}x. \tag{68}$$

From this inequality we can derive lower and upper bounds for the value of the sum $1/\sqrt{1} + 1/\sqrt{2} + \cdots + 1/\sqrt{N}$, i.e.,

$$\int_{1}^{N+1} \frac{1}{\sqrt{x}}\,\mathrm{d}x = \sum_{k=1}^{N} \int_{k}^{k+1} \frac{1}{\sqrt{x}}\,\mathrm{d}x < \sum_{k=1}^{N} \frac{1}{\sqrt{k}} < \sum_{k=1}^{N} \int_{k-1}^{k} \frac{1}{\sqrt{x}}\,\mathrm{d}x = \int_{0}^{N} \frac{1}{\sqrt{x}}\,\mathrm{d}x. \tag{69}$$

The left-most integral evaluates to:

$$\int_{1}^{N+1} \frac{1}{\sqrt{x}}\,\mathrm{d}x = 2\sqrt{x}\,\Big|_{1}^{N+1} = 2\sqrt{N+1} - 2. \tag{70}$$

Similarly, the right-most integral has the following value:

$$\int_{0}^{N} \frac{1}{\sqrt{x}}\,\mathrm{d}x = 2\sqrt{x}\,\Big|_{0}^{N} = 2\sqrt{N}. \tag{71}$$

Combining (69) with (70) and (71) leads to:

$$2\sqrt{N+1} - 2 < \sum_{k=1}^{N} \frac{1}{\sqrt{k}} < 2\sqrt{N}, \tag{72}$$

from which (67) follows. □

S5. Proof of Formula (2)

This section proves formula (2), which expresses the length of the Farey sequence F_n recursively in terms of the lengths of the Farey sequences of lower orders. This formula is well-known, but its proof is hard to find. Our proof uses only basic algebra and mathematical induction. The proof also uses Euler's totient function $\varphi(n)$, which is defined in Section S3.

Theorem 12. *The length of the Farey sequence of order n can be computed recursively as follows:*

$$|F_n| = \frac{(n+3)n}{2} - \sum_{k=2}^{n} \left|F_{\lfloor n/k \rfloor}\right|, \tag{73}$$

where $\lfloor x \rfloor$ denotes the largest integer that does not exceed x.

Proof. The proof is by mathematical induction. The base case of the induction is formed by the length of F_n when $n = 1$, i.e.,

$$|F_1| = \frac{(1+3)\cdot 1}{2} = 2. \tag{74}$$

To prove the inductive step, we will introduce the term T_n that is defined as follows:

$$T_n = |F_n| + \sum_{k=2}^{n} \left|F_{\lfloor n/k \rfloor}\right|. \tag{75}$$

Our goal is to prove that

$$T_n = \frac{(n+3)n}{2}. \tag{76}$$

We have already established that this is true for $n = 1$. Assuming that this formula holds for some $n \geq 1$, our next goal is to prove that it also holds for T_{n+1}. In other words, we would like to show that the value of T_{n+1} can be expressed as follows:

$$\begin{aligned} T_{n+1} &= |F_{n+1}| + \sum_{k=2}^{n+1} \left|F_{\lfloor (n+1)/k \rfloor}\right| \\ &= \frac{((n+1)+3)(n+1)}{2} \\ &= \frac{(n+3)n}{2} + \frac{2n+4}{2} \\ &= T_n + n + 2. \end{aligned} \tag{77}$$

Using Theorem 1 and Definition 1 (see Section S3), we can express the term T_{n+1} as shown below:

$$\begin{aligned} T_{n+1} &= |F_{n+1}| \; + \sum_{k=2}^{n+1} \left|F_{\left\lfloor \frac{n+1}{k} \right\rfloor}\right| \\ &= |F_n| + \varphi(n+1) \; + \sum_{k=2}^{n+1} \left|F_{\left\lfloor \frac{n+1}{k} \right\rfloor}\right| \\ &= |F_n| + \varphi(n+1) + \sum_{k=2}^{n} \left|F_{\left\lfloor \frac{n+1}{k} \right\rfloor}\right| + \underbrace{\sum_{k=n+1}^{n+1} \left|F_{\left\lfloor \frac{n+1}{k} \right\rfloor}\right|}_{|F_1|} \\ &= |F_n| + \varphi(n+1) + \sum_{k=2}^{n} \left|F_{\left\lfloor \frac{n+1}{k} \right\rfloor}\right| + 2. \end{aligned} \tag{78}$$

Lemmas 6 and 7 (see Section S4) imply that in (78) the value of each term in the sum from 2 to n can be expressed as follows:

$$\left|F_{\left\lfloor \frac{n+1}{k} \right\rfloor}\right| = \begin{cases} \left|F_{\left\lfloor \frac{n}{k} \right\rfloor}\right|, & \text{if } (n+1) \bmod k \neq 0, \\ \left|F_{\left\lfloor \frac{n}{k} \right\rfloor + 1}\right|, & \text{if } (n+1) \bmod k = 0. \end{cases} \tag{79}$$

Moreover, for the second case in (79), Theorem 1 and Lemma 7 imply that:

$$\left|F_{\left\lfloor \frac{n+1}{k} \right\rfloor}\right| = \left|F_{\left\lfloor \frac{n}{k} \right\rfloor + 1}\right| = \left|F_{\left\lfloor \frac{n}{k} \right\rfloor}\right| + \varphi\left(\frac{n+1}{k}\right), \qquad \text{if } (n+1) \bmod k = 0. \tag{80}$$

Let D be the set of all positive integer divisors of $n{+}1$ that lie between 2 and n. That is,

$$D = \big\{k \in \{2, 3, \ldots, n\} \text{ s.t. } (n+1) \bmod k = 0\big\}. \tag{81}$$

Then, the sum in equation (78) can be expressed as follows:

$$\sum_{k=2}^{n} \left|F_{\left\lfloor \frac{n+1}{k} \right\rfloor}\right| = \sum_{k=2}^{n} \left|F_{\left\lfloor \frac{n}{k} \right\rfloor}\right| + \sum_{k \in D} \varphi\left(\frac{n{+}1}{k}\right). \tag{82}$$

If an integer k is an element of the set D, then the value of $(n{+}1)/k$ is also an element of D and vice versa. Therefore,

$$\sum_{k \in D} \varphi\left(\frac{n{+}1}{k}\right) = \sum_{k \in D} \varphi(k). \tag{83}$$

Thus, equation (82) can be restated as:

$$\sum_{k=2}^{n} \left|F_{\left\lfloor \frac{n+1}{k} \right\rfloor}\right| = \sum_{k=2}^{n} \left|F_{\left\lfloor \frac{n}{k} \right\rfloor}\right| + \sum_{k \in D} \varphi(k). \tag{84}$$

Plugging this result into (78) leads to the following formula for the value of T_{n+1}:

$$T_{n+1} = |F_n| + \varphi(n{+}1) + \sum_{k=2}^{n} \left|F_{\left\lfloor \frac{n}{k} \right\rfloor}\right| + \sum_{k \in D} \varphi(k) + 2. \tag{85}$$

Because $\varphi(1) = 1$, it follows that $\varphi(1) + 1 = 2$. This allows us to rearrange the terms in the last equation as follows:

$$\begin{aligned} T_{n+1} &= \underbrace{|F_n| + \sum_{k=2}^{n} \left|F_{\left\lfloor \frac{n}{k} \right\rfloor}\right|}_{T_n} + \underbrace{\varphi(n{+}1) + \sum_{k \in D} \varphi(k) + \varphi(1)}_{\sum_{k \mid n+1} \varphi(k)} + 1 \\ &= T_n + \sum_{k \mid n+1} \varphi(k) \, + \, 1. \end{aligned} \tag{86}$$

In this formula, $\sum_{k \mid n+1} \varphi(k)$ denotes the sum of the values of Euler's totient function $\varphi(k)$ for all k that are positive integer divisors of $n+1$. In 1798, Gauss proved[14] that this sum is equal to $n{+}1$. Thus,

$$T_{n+1} = T_n + (n+1) + 1 = T_n + n + 2, \tag{87}$$

which proves equation (77) as required. □

S6. Proof of Formula (5)

This section proves another recursive formula for the value of $|F_n|$. This formula is derived from formula (2) by splitting the sum into two segments and grouping the repeated terms in the second segment. The resulting formula is longer, but it needs to add fewer terms. It leads to Algorithm 5 (i.e., FareyLengthC), which is described in the main paper.

Theorem 13. *For each $n > 1$, the length of the Farey sequence F_n can be expressed using the following recursive formula:*

$$|F_n| = \frac{(n+3)n}{2} - \sum_{k=2}^{u(n)} \left|F_{\lfloor n/k \rfloor}\right| - \sum_{k=1}^{\lfloor\sqrt{n}\rfloor} \left(\left\lfloor \frac{n}{k} \right\rfloor - \left\lfloor \frac{n}{k+1} \right\rfloor \right) \cdot |F_k|, \tag{88}$$

where $u(n) = \left\lfloor \frac{n}{\lfloor\sqrt{n}\rfloor+1} \right\rfloor$.

Proof. This formula is derived from formula (2), which is replicated below:

$$|F_n| = \frac{(n+3)n}{2} - \sum_{k=2}^{n} \left|F_{\lfloor n/k \rfloor}\right| . \tag{89}$$

The first step is to split the summation over k at $u(n)$ into two separate sums:

$$\sum_{k=2}^{n} \left|F_{\lfloor n/k \rfloor}\right| = \sum_{k=2}^{u(n)} \left|F_{\lfloor n/k \rfloor}\right| + \sum_{k=u(n)+1}^{n} \left|F_{\lfloor n/k \rfloor}\right| . \tag{90}$$

And then change the index variable from k to m in the second sum:

$$\sum_{k=2}^{n} \left|F_{\lfloor n/k \rfloor}\right| = \sum_{k=2}^{u(n)} \left|F_{\lfloor n/k \rfloor}\right| + \sum_{m=u(n)+1}^{n} \left|F_{\lfloor n/m \rfloor}\right| . \tag{91}$$

The next step is to express the last sum in formula (91) so that it has only $\lfloor\sqrt{n}\rfloor$ terms. Lemma 4 proves that the set of the indices m used in that sum is equal to the following union:

$$\bigcup_{k=1}^{\lfloor\sqrt{n}\rfloor} S(k,n) = \{u(n)+1,\, u(n)+2, \ldots,\, n\}, \tag{92}$$

where each set $S(k,n)$ consists of all positive integers m for which $\lfloor n/m \rfloor = k$, i.e.,

$$S(k,n) = \left\{ \left\lfloor \frac{n}{k+1} \right\rfloor + 1, \left\lfloor \frac{n}{k+1} \right\rfloor + 2, \ldots, \left\lfloor \frac{n}{k} \right\rfloor \right\}. \tag{93}$$

Lemma 4 also proves that all sets $S(k,n)$ in this union are disjoint. Each of these sets has $\left\lfloor \frac{n}{k} \right\rfloor - \left\lfloor \frac{n}{k+1} \right\rfloor$ elements. Therefore, for each $n > 1$ the last sum in (91) can be expressed as follows:

$$\sum_{m=u(n)+1}^{n} \left|F_{\lfloor n/m \rfloor}\right| = \sum_{k=1}^{\lfloor\sqrt{n}\rfloor} \sum_{m \in S(k,n)} |F_k| = \sum_{k=1}^{\lfloor\sqrt{n}\rfloor} \left(\left\lfloor \frac{n}{k} \right\rfloor - \left\lfloor \frac{n}{k+1} \right\rfloor \right) |F_k|. \tag{94}$$

Substituting this expression into (91) and that result into (89) proves the theorem. □

Formula (88) cannot express F_1 because the last sum leads to an infinite recursion when $n = 1$. Also, in this special case the sum in formula (89) is degenerate. In contrast to the cases when $n \geq 2$, the sum cannot be split in a way that leads to formula (90) when $n = 1$. Therefore, any algorithm that implements formula (88) should handle $n = 1$ as a special case. For example, F_1 can be hard-coded to be equal to 2.

S7. Computing $\lfloor\sqrt{n}\rfloor$ and $\lfloor\sqrt[3]{n^2}\rfloor$ exactly

Algorithms S4 and S5 give the pseudo-code for two helper functions that are used by several of the algorithms. The first function computes $\lfloor\sqrt{n}\rfloor$ exactly using Newton's method and integer arithmetic.[28] The second function computes the value of $\lfloor\sqrt[3]{n}\rfloor$ exactly using a similar approach. All algorithms, however, call ICBRT with an argument that is equal to n^2 instead of n. Thus, it is used to compute $\lfloor\sqrt[3]{n^2}\rfloor$. Both algorithms run in $O(\log\log n)$ time and use $O(1)$ memory.

Algorithm S4. Compute $\lfloor\sqrt{n}\rfloor$ exactly using integer arithmetic. Runs in $O(\log\log n)$ time and uses $O(1)$ memory.

function ISQRT(n)
    x $\leftarrow n$;
    y $\leftarrow \lceil n/2 \rceil$;
    while y $<$ x **do**
        x $\leftarrow$ y;
        y $\leftarrow \left\lfloor \frac{x + \lfloor n/x \rfloor}{2} \right\rfloor$;
    end while
    return x;
end function

Algorithm S5. Compute $\lfloor\sqrt[3]{n}\rfloor$ exactly using integer arithmetic. Runs in $O(\log\log n)$ time and uses $O(1)$ memory.

function ICBRT(n)
    x $\leftarrow n$;
    y $\leftarrow \lceil n/3 \rceil$;
    while y $<$ x **do**
        x $\leftarrow$ y;
        y $\leftarrow \left\lfloor \frac{2x + \lfloor n/x^2 \rfloor}{3} \right\rfloor$;
    end while
    return x;
end function

S8. Lookup Table Design

Some of the algorithms described in this paper use a lookup table called F to store the values of $|F_m|$ for different m. The computational complexity estimates assume that each entry in this lookup table can be accessed in $O(1)$ time. They also assume that storing a lookup table of size N requires $O(N)$ memory.

This appendix describes an implementation for a lookup table that satisfies these time and space complexity constraints. The implementation is specific to our algorithms, i.e., it works for the special case when all keys (or indices) that are used are in the set $\{1, 2, \ldots, d\} \cup \left\{\left\lfloor\frac{n}{\lfloor n/d\rfloor}\right\rfloor, \ldots, \left\lfloor\frac{n}{2}\right\rfloor, \left\lfloor\frac{n}{1}\right\rfloor\right\}$. Alternatively, it is possible to use a generic hash table as a lookup table (e.g., the hash table implemented in the standard dictionary class in the Python language). In that case the computational complexity of setting or getting entries is in $O(1)$ on average. Even though this is a minor technical point, this appendix shows that the desired performance can be achieved in all cases, i.e., not just on average.

Algorithm S6 gives the pseudo-code for initializing the lookup table. The function has two parameters: n and d. The value of n determines the size of the problem, i.e., the order of the Farey sequence F_n for which we want to compute the length. The parameter d controls the split between the two sets of keys that the table supports. The table is organized as two arrays. The first array, X, stores the entries for the keys in the set $\{1, 2, \ldots, d\}$. The second array, Y, stores the entries for the keys in the set $\left\{\left\lfloor\frac{n}{\lfloor n/d\rfloor}\right\rfloor, \ldots, \left\lfloor\frac{n}{2}\right\rfloor, \left\lfloor\frac{n}{1}\right\rfloor\right\}$. In most cases, $d = \lfloor\sqrt{n}\rfloor + 1$, except for Algorithm S13, where it is set to $\lfloor\sqrt[3]{n^2}\rfloor + 1$.

Algorithm S7 gives the pseudo-code for getting the value of an element from the lookup table, given a key k. This function is called when the square bracket notation is used with F in our algorithms, i.e., $F[k]$ translates to GetItem(F, k);

Algorithm S8 gives the pseudo-code for setting an element of the lookup table. Once again, it is assumed that an assignment $F[k] \leftarrow x$ in our algorithms translates to a call to SetItem(F, k, x).

Finally, Algorithm S9 gives the pseudo-code for a function that checks if the value for the key k is set in the lookup table F. This check is used in some of our algorithms for diagnostic purposes. That is, it is assumed that a check k **in** F translates to a function call Contains(F, k).

Algorithm S6. Initialize a lookup table. Uses $O(d + n/d)$ memory.

```
function LookupTable(n, d)
    F ← NewObject();
    F.n ← n;
    F.X ← NullArray(d);
    F.Y ← NullArray(⌊n/d⌋ + 1);
    return F;
end function
```

Algorithm S7. Get the value for a key from the lookup table. Runs in $O(1)$ time.

```
function GetItem(F, k)
    if k < Length(F.X) then
        return F.X[k];
    else
        return F.Y[⌊F.n/k⌋];
    end if
end function
```

Algorithm S8. Set the value for a key in the lookup table. Runs in $O(1)$ time.

```
function SetItem(F, k, x)
    if k < Length(F.X) then
        F.X[k] ← x;
    else
        F.Y[⌊F.n/k⌋] ← x;
    end if
end function
```

Algorithm S9. Check if the value for a key is set in the lookup table. Runs in $O(1)$ time.

```
function Contains(F, k)
    if k < Length(F.X) then
        return (F.X[k] is not null);
    else
        m ← ⌊F.n/k⌋;
        if k ≠ ⌊F.n/m⌋ then
            return False;
        end if
        return (F.Y[m] is not null);
    end if
end function
```

S9. Two algorithms that run in $O(n \log n)$ time and use $O(\sqrt{n})$ memory

This section describes two $O(n \log n)$ algorithms for computing the length of the Farey sequence of order n. The algorithms are based on a combination of formulas (2) and (5) from the main paper, which are reproduced below:

$$|F_n| = \frac{(n+3)\,n}{2} - \sum_{k=2}^{n} \left|F_{\lfloor n/k \rfloor}\right|, \tag{95}$$

$$|F_n| = \frac{(n+3)\,n}{2} - \sum_{k=2}^{u(n)} \left|F_{\lfloor n/k \rfloor}\right| - \sum_{k=1}^{\lfloor \sqrt{n} \rfloor} \left(\left\lfloor \frac{n}{k} \right\rfloor - \left\lfloor \frac{n}{k+1} \right\rfloor \right) \cdot |F_k|. \tag{96}$$

Algorithm S10 gives the pseudo-code for the first algorithm, which is given a suffix letter L. The helper function in this case implements formula (95). The main algorithm, however, structures the calls to this function in the same way as Algorithm 5, which is based on formula (96). Theorem 14 (see below) proves that the computational complexity of this algorithm is in $O(n \log n)$. The space complexity is in $O(\sqrt{n})$. This is easy to show because FAREYLENGTHL is structured similarly to FAREYLENGTHC, except for using a different helper function (see also the proofs in Section S10).

Algorithm S10. Compute the length of the Farey sequence F_n. Runs in $O(n \log n)$ time and uses $O(\sqrt{n})$ memory.

```
function FAREYLENGTHL(n)
    r ← ISQRT(n);
    u ← ⌊n/(r+1)⌋;
    F ← LOOKUPTABLE(n, r + 1);
    F[1] ← 2;
    for m ← 2 to r do
        UPDATELOOKUPTABLEL(F, m);
    end for
    for j ← u down to 1 do
        UPDATELOOKUPTABLEL(F, ⌊n/j⌋);
    end for
    return F[n];
end function

function UPDATELOOKUPTABLEL(F, m)        // helper function
    s ← 0;
    for k ← 2 to m do
        q ← ⌊m/k⌋;
        ASSERT(q in F);
        s ← s + F[q];
    end for
    F[m] ← (m + 3) m / 2 − s;
end function
```

Algorithm S11 is a recursive version of Algorithm S10. This algorithm also runs in $O(n \log n)$ time and uses $O(\sqrt{n})$ memory. This can be established by unpacking the recursion, which leads to the iterative version presented above. This is the shortest algorithm described in this paper. Unfortunately, it is also one of the slowest. For small values of n, however, it could be very useful. For example, it can be used to verify the output of the faster algorithms.

Algorithm S11. Recursive algorithm for computing the length of F_n. Runs in $O(n \log n)$ time and uses $O(\sqrt{n})$ memory.

```
function FAREYLENGTHLR(n, F = null)
    if F is null then
        F ← LOOKUPTABLE(n, ISQRT(n) + 1);        // initialize the lookup table F for memoization
    end if
    if n in F then
        return F[n];
    end if
    s ← 0;
    for k ← 2 to n do
        s ← s + FAREYLENGTHLR(⌊n/k⌋, F);
    end for
    F[n] ← (n + 3) n / 2 − s;                    // update the lookup table
    return F[n];
end function
```

Theorem 14. *Algorithm S10 (i.e.,* FAREYLENGTHL*) runs in $O(n \log n)$ time.*

Proof. One call to the helper function UPDATELOOKUPTABLEL computes exactly one new entry in the lookup table F. The main algorithm calls this function in the correct order such that the elements of F that the helper function uses are already computed by the time it is called. Thus, the computational complexity of the helper function is in $O(m)$, because it has only one loop that runs for $m-1$ iterations in order to compute $F[m]$.

The main algorithm calls the helper function from within two for-loops, but they are not nested. Therefore, the computational complexity of the algorithm can be expressed as follows:

$$\sum_{m=2}^{\lfloor\sqrt{n}\rfloor} O(m) + \sum_{j=1}^{u(n)} O(\lfloor n/j \rfloor) = O\left(\sum_{m=2}^{\lfloor\sqrt{n}\rfloor} m\right) + O\left(\sum_{j=1}^{u(n)} \lfloor n/j \rfloor\right). \tag{97}$$

The first sum in the right-hand side of this formula is in $O(n)$. That is,

$$\sum_{m=2}^{\lfloor\sqrt{n}\rfloor} m = \frac{(\lfloor\sqrt{n}\rfloor - 1)(\lfloor\sqrt{n}\rfloor + 2)}{2} = \frac{n}{2} + \frac{\lfloor\sqrt{n}\rfloor}{2} - 1 = O(n). \tag{98}$$

The second sum in the right-hand side of (97) is in $O(n \log n)$. This can be shown as follows:

$$\sum_{j=1}^{u(n)} \lfloor n/j \rfloor \leq \sum_{j=1}^{\lfloor\sqrt{n}\rfloor} \lfloor n/j \rfloor \leq \sum_{j=1}^{\lfloor\sqrt{n}\rfloor} \frac{n}{j} = n \left(\sum_{j=1}^{\lfloor\sqrt{n}\rfloor} \frac{1}{j}\right). \tag{99}$$

In this formula, the left-most inequality follows from Lemma 5, which implies that $u(n) \leq \lfloor\sqrt{n}\rfloor$. The derivation can be continued using Lemma 10, which implies that:

$$\sum_{j=1}^{\lfloor\sqrt{n}\rfloor} \frac{1}{j} = O\big(\log\lfloor\sqrt{n}\rfloor\big) = O(\log n). \tag{100}$$

Therefore,

$$\sum_{j=1}^{u(n)} \lfloor n/j \rfloor = O(n \log n). \tag{101}$$

Combining formulas (98) and (101) with formula (97) proves that Algorithm S10 runs in $O(n \log n)$ time. □

S10. Computational Complexity Analysis of FareyLengthC

This section analyzes the computational complexity of FareyLengthC (i.e., Algorithm 5) and proves that it runs in $O(n^{3/4})$ time and uses $O(\sqrt{n})$ memory. It also proves that the elements of the lookup table are computed in such a way that they are always available when the helper function needs them.

The following theorem gives the time complexity of the helper function UpdateLookupTable (i.e., Algorithm 6).

Theorem 15. *Algorithm 6 runs in $O(\sqrt{m})$ time.*

Proof. This algorithm implements a helper function that uses formula (5) to compute the value of F_m. That is,

$$|F_m| = \frac{(m+3)m}{2} - \sum_{k=2}^{u(m)} \left|F_{\lfloor m/k \rfloor}\right| - \sum_{k=1}^{\lfloor \sqrt{m} \rfloor} \left(\left\lfloor \frac{m}{k} \right\rfloor - \left\lfloor \frac{m}{k+1} \right\rfloor \right) \cdot |F_k|. \tag{102}$$

This function has two for-loops, but they are not nested. These loops implement the first and the second summation in the formula, respectively. One call to this function computes exactly one new entry in the lookup table F. The main algorithm (i.e., Algorithm 5) ensures that all calls to this function are performed in the correct order such that the values from F that it uses are already set because they were computed earlier (see the proofs on the next page).

The computational complexity of each iteration in either of the two loops is in $O(1)$. Thus, the computational complexity of the helper function can be determined as follows:

$$O\left(\sum_{k=2}^{u(m)} 1 + \sum_{k=1}^{\lfloor \sqrt{m} \rfloor} 1 \right) = O\big(u(m) - 1 + \lfloor \sqrt{m} \rfloor \big) = O\big(\sqrt{m}\big) . \tag{103}$$

This result follows from Lemma 5, which proves that $u(m) \leq \lfloor \sqrt{m} \rfloor$. This, in turn, implies that $u(m) - 1 + \lfloor \sqrt{m} \rfloor < 2\lfloor \sqrt{m} \rfloor$. Therefore, this function runs in $O(\sqrt{m})$ time, where m is its argument, i.e., not the n for which the main algorithm is called. ☐

The next theorem establishes the time and space complexity of FareyLengthC (i.e., Algorithm 5).

Theorem 16. *Algorithm 5 runs in $O(n^{3/4})$ time and uses $O(\sqrt{n})$ memory.*

Proof. The algorithm calls the helper function from inside two for-loops, which are not nested. The first loop iterates over m from 2 to $\lfloor \sqrt{n} \rfloor$. The second loop iterates over j from $u(n)$ down to 1. Thus, the overall computational complexity can be expressed as follows:

$$\sum_{m=2}^{\lfloor \sqrt{n} \rfloor} O\big(\sqrt{m}\big) + \sum_{j=1}^{u(n)} O\left(\sqrt{\left\lfloor \frac{n}{j} \right\rfloor} \right) = O\left(\sum_{m=2}^{\lfloor \sqrt{n} \rfloor} \sqrt{m} \right) + O\left(\sum_{j=1}^{u(n)} \sqrt{\left\lfloor \frac{n}{j} \right\rfloor} \right) . \tag{104}$$

The first sum in the right-hand side of (104) can be bounded from above as follows:

$$\sum_{m=2}^{\lfloor \sqrt{n} \rfloor} \sqrt{m} \leq \sum_{m=2}^{\lfloor \sqrt{n} \rfloor} \sqrt{\lfloor \sqrt{n} \rfloor} \leq \sum_{m=2}^{\lfloor \sqrt{n} \rfloor} \sqrt{\sqrt{n}} < \lfloor \sqrt{n} \rfloor \sqrt{\sqrt{n}} \leq \sqrt{n}\sqrt{\sqrt{n}} = n^{1/2}\, n^{1/4} = n^{3/4}. \tag{105}$$

This result implies that

$$O\left(\sum_{m=2}^{\lfloor \sqrt{n} \rfloor} \sqrt{m} \right) = O(n^{3/4}). \tag{106}$$

The second sum in the right-hand side of (104) can also be bounded from above:

$$\sum_{j=1}^{u(n)} \sqrt{\left\lfloor \frac{n}{j} \right\rfloor} \leq \sum_{j=1}^{\lfloor \sqrt{n} \rfloor} \sqrt{\left\lfloor \frac{n}{j} \right\rfloor} \leq \sum_{j=1}^{\lfloor \sqrt{n} \rfloor} \sqrt{\frac{n}{j}} = \sqrt{n} \sum_{j=1}^{\lfloor \sqrt{n} \rfloor} \frac{1}{\sqrt{j}} = \sqrt{n}\, O\big(\sqrt{\lfloor \sqrt{n} \rfloor}\big) = O(n^{3/4}), \tag{107}$$

which follows from Lemmas 5 and 11.

To summarize, we showed that both terms in the right-hand side of (104) are in $O(n^{3/4})$. Therefore, the computational complexity of FareyLengthC is also in $O(n^{3/4})$. The algorithm fills $\lfloor \sqrt{n} \rfloor$ entries of the lookup table F in the first for-loop and $u(n)$ entries in the second for-loop. Because $u(n) \leq \lfloor \sqrt{n} \rfloor$, the space complexity of the algorithm is in $O(\sqrt{n})$. ☐

Algorithm 5 uses the helper function to compute the value of $|F_m|$ for each integer m in two lists. The first list has $\lfloor\sqrt{n}\rfloor - 1$ elements and consists of the numbers $2, 3, \ldots, \lfloor\sqrt{n}\rfloor$. The second list has $u(n)$ elements and consists of the numbers $\left\lfloor \frac{n}{u(n)} \right\rfloor$, $\left\lfloor \frac{n}{u(n)-1} \right\rfloor$, ..., $\left\lfloor \frac{n}{1} \right\rfloor$. The algorithm processes the first list sequentially starting from 2. After that, it processes the second list sequentially starting from $\lfloor n/u(n)\rfloor$. In total, the algorithm processes $\lfloor\sqrt{n}\rfloor - 1 + u(n) \leq 2\lfloor\sqrt{n}\rfloor$ values of m.

Let $Q(m)$ be the set of keys in the lookup table F that are used by the first for-loop of the helper function (i.e., Algorithm 6). For a given value of m this set is equal to:

$$Q(m) = \left\{ \left\lfloor \frac{m}{2} \right\rfloor, \left\lfloor \frac{m}{3} \right\rfloor, \ldots, \left\lfloor \frac{m}{u(m)} \right\rfloor \right\}. \tag{108}$$

Let $K(m)$ be the set of keys in the lookup table F that are used by the second for-loop of the helper function. That is,

$$K(m) = \{1, 2, \ldots, \lfloor\sqrt{m}\rfloor\}. \tag{109}$$

The following theorem helps us to prove that all entries in the lookup table F that the helper function uses to compute the value of $F[m]$ are already set when the function is called in the first for-loop of Algorithm 5.

Theorem 17. *Let m be an integer that is greater than 1. Then, both $Q(m)$ and $K(m)$ are subsets of the set of all integers in the interval $[1, m-1]$. More formally,*

$$Q(m) \subseteq \{1, 2, \ldots, m-1\}, \tag{110}$$

$$K(m) \subseteq \{1, 2, \ldots, m-1\}. \tag{111}$$

Proof. The elements of the set $Q(m)$ fall between 1 and $\lfloor m/2\rfloor$, which does not exceed $m-1$. The reason for this is that $u(m) \geq 2$ when $m \geq 2$, i.e., the maximum element of $Q(m)$ is $\lfloor m/2\rfloor$. The minimum element is equal to $\lfloor m/u(m)\rfloor$, which is a positive integer. Thus, $Q(m) \subseteq \{1, 2, \ldots, m-1\}$.

The elements of the set $K(m)$ fall between 1 and $\lfloor\sqrt{m}\rfloor$. Also, the value of $\lfloor\sqrt{m}\rfloor$ does not exceed $m-1$ for $m \geq 2$. Therefore, $K(m) \subseteq \{1, 2, \ldots, m-1\}$, as required. □

Corollary 18. *All entries in the lookup table F that are required for computing $F[m]$ during each iteration of the first for-loop in* FAREYLENGTHC *have already been computed.*

Proof. This follows from Theorem 17. The algorithm sets $F[1]$ to 2 and then loops over the values of m in the list $(2, 3, \ldots, \lfloor\sqrt{n}\rfloor)$ in increasing order. Thus, when the algorithm reaches the iteration that computes $F[m]$, the lookup table already contains the entries for the keys in the set $\{1, 2, \ldots, m-1\}$. Therefore, Theorem 17 implies that all prerequisite elements for computing $F[m]$ are already computed when the helper function is called to calculate it. □

Let $Y(j)$ be the set of keys in the lookup table F that are already computed by Algorithm 5 before it calls the helper function in the second for-loop with the parameter m equal to $\lfloor n/j\rfloor$. More formally,

$$Y(j) = \{1, 2, \ldots, \lfloor\sqrt{n}\rfloor\} \cup \left\{ \left\lfloor \frac{n}{u(n)} \right\rfloor, \left\lfloor \frac{n}{u(n)-1} \right\rfloor, \ldots, \left\lfloor \frac{n}{j+1} \right\rfloor \right\}. \tag{112}$$

Then, the following theorem helps us to prove that the second for-loop in Algorithm 5 also uses only those entries of F that have already been set.

Theorem 19. *Let n be a positive integer and let j be an integer between 1 and $u(n)$. Then,*

$$K(\lfloor n/j\rfloor) \subseteq Y(j), \tag{113}$$

$$Q(\lfloor n/j\rfloor) \subseteq Y(j). \tag{114}$$

Proof. Clearly, formula (113) holds because

$$K(\lfloor n/j\rfloor) = \left\{1, 2, \ldots, \left\lfloor\sqrt{n/j}\right\rfloor\right\} \subseteq \{1, 2, \ldots, \lfloor\sqrt{n}\rfloor\} \subseteq Y(j). \tag{115}$$

To prove formula (114), suppose that $k \in Q(\lfloor n/j\rfloor)$. Then, there is an integer q such that $q \in \{2, 3, \ldots, u(\lfloor n/j\rfloor)\}$ and $k = \left\lfloor \frac{\lfloor n/j\rfloor}{q} \right\rfloor$. Lemma 8 implies that $k = \left\lfloor \frac{n}{jq} \right\rfloor$. If $k \leq \lfloor\sqrt{n}\rfloor$, then $k \in Y(j)$ because, by definition, $Y(j)$ includes all integers between 1 and $\lfloor\sqrt{n}\rfloor$. If $k > \lfloor\sqrt{n}\rfloor$, then $k \geq \lfloor\sqrt{n}\rfloor + 1$, which implies that $\frac{n}{jq} \geq \lfloor\sqrt{n}\rfloor + 1$. Therefore, $jq \leq \frac{n}{\lfloor\sqrt{n}\rfloor+1}$. Because the value of the product jq is an integer, $jq \leq \left\lfloor \frac{n}{\lfloor\sqrt{n}\rfloor+1} \right\rfloor = u(n)$. Also, from $q \geq 2$ it follows that $jq > j$. Therefore, $jq \in \{j+1, j+2, \ldots, u(n)\}$, and, thus,

$$\left\lfloor \frac{n}{jq} \right\rfloor \in \left\{ \left\lfloor \frac{n}{u(n)} \right\rfloor, \left\lfloor \frac{n}{u(n)-1} \right\rfloor, \ldots, \left\lfloor \frac{n}{j+1} \right\rfloor \right\}. \tag{116}$$

This implies that $k \in Y(j)$, which shows that $Q(\lfloor n/j\rfloor) \subseteq Y(j)$ and completes the proof of the theorem. □

Corollary 20. *For each iteration of the second for-loop in Algorithm 5, all entries in the lookup table F that are necessary for computing the value of $\left|F_{\lfloor n/j \rfloor}\right|$ are already available when the helper function is called to compute it.*

Proof. This corollary follows from Theorem 19. After the end of the first for-loop of Algorithm 5 the values of $|F_m|$ for $m \in \{1, 2, \ldots, \lfloor\sqrt{n}\rfloor\}$ are already computed. The second for-loop iterates over values of j from the list $(u(n), u(n)-1, \ldots, 1)$ in decreasing order starting from $u(n)$. Thus, the entries for keys from the set $Y(j)$ are already set when the control flow enters the helper function with $m = \lfloor n/j \rfloor$. The function uses keys from the sets $Q(\lfloor n/j \rfloor)$ and $K(\lfloor n/j \rfloor)$ to compute $\left|F_{\lfloor n/j \rfloor}\right|$. Because both $Q(\lfloor n/j \rfloor)$ and $K(\lfloor n/j \rfloor)$ are subsets of $Y(j)$, all required entries are available and the helper function can compute $\left|F_{\lfloor n/j \rfloor}\right|$ successfully. ☐

S11. Recursive version of FareyLengthC

Algorithm S12 gives the pseudo-code for a recursive version of Algorithm 5 (i.e., FareyLengthC). This version is a direct implementation of formula (5), which is replicated below:

$$|F_n| = \frac{(n+3)n}{2} - \sum_{k=2}^{u(n)} \left|F_{\lfloor n/k \rfloor}\right| - \sum_{k=1}^{\lfloor\sqrt{n}\rfloor} \left(\left\lfloor \frac{n}{k} \right\rfloor - \left\lfloor \frac{n}{k+1} \right\rfloor \right) \cdot |F_k|. \tag{117}$$

In this case, however, the required values of $|F_k|$ and $|F_{\lfloor n/k \rfloor}|$ are computed with recursive calls. These intermediate values are stored in the lookup table F. Similarly to the iterative version, this algorithm also runs in $O(n^{3/4})$ time and uses $O(\sqrt{n})$ memory. This can be shown by unpacking the recursive calls, which leads to the iterative version described in the main text.

Algorithm S12. Recursive algorithm for the length of the Farey sequence F_n. Runs in $O(n^{3/4})$ time and uses $O(\sqrt{n})$ memory.

function FareyLengthCR(n, F = **null**)
  $r \leftarrow$ ISQRT(n); // call Algorithm S4 to compute $\lfloor\sqrt{n}\rfloor$ exactly
  $u \leftarrow \left\lfloor \frac{n}{r+1} \right\rfloor$;
  if F **is null then**
    $F \leftarrow$ LookupTable(n, $r+1$); // initialize the lookup table F for memoization
    $F[1] \leftarrow 2$; // set $F[1]$ to $|F_1| = 2$
  end if
  if n **in** F **then**
    return $F[n]$;
  end if
  $s \leftarrow 0$;
  for $k \leftarrow 2$ **to** u **do**
    $s \leftarrow s +$ FareyLengthCR$\left(\left\lfloor \frac{n}{k} \right\rfloor, F\right)$;
  end for
  for $k \leftarrow 1$ **to** r **do**
    $s \leftarrow s + \left(\left\lfloor \frac{n}{k} \right\rfloor - \left\lfloor \frac{n}{k+1} \right\rfloor\right) \cdot$ FareyLengthCR(k, F);
  end for
  $F[n] \leftarrow \dfrac{(n+3)\,n}{2} - s$; // update the lookup table
  return $F[n]$;
end function

S12. Computational Complexity Analysis of FareyLengthD

FareyLengthD can be viewed as a mixture between FareyLengthA and FareyLengthC. It picks an optimal point to divide the computation between these two algorithms as described below.

In order to compute $|F_n|$, FareyLengthC (i.e., Algorithm 5) computes $|F_m|$ for values of m that can be split into two sets M_1 and M_2 that correspond to the two for-loops in the algorithm. That is, the first for-loop computes the length of F_m for $m \in M_1$ and the second for-loop computes the length of F_m for $m \in M_2$. The set M_1 includes all integers between 1 and $\lfloor\sqrt{n}\rfloor$, i.e.,

$$M_1 = \{1, 2, \ldots, \lfloor\sqrt{n}\rfloor\}. \tag{118}$$

The set M_2 is defined by the following formula:

$$M_2 = \left\{ \left\lfloor \tfrac{n}{u(n)} \right\rfloor, \left\lfloor \tfrac{n}{u(n)-1} \right\rfloor, \ldots, \left\lfloor \tfrac{n}{2} \right\rfloor, \left\lfloor \tfrac{n}{1} \right\rfloor \right\}. \tag{119}$$

Because $u(n) = \left\lfloor \frac{n}{\lfloor\sqrt{n}\rfloor+1} \right\rfloor$, it follows that $\left\lfloor \frac{n}{u(n)} \right\rfloor \geq \lfloor\sqrt{n}\rfloor + 1$. Thus, each element of M_2 falls between $\lfloor\sqrt{n}\rfloor + 1$ and n. This set is sparse because it has $u(n)$ elements, but the total number of integers between $\lfloor\sqrt{n}\rfloor + 1$ and n is equal to $n - \lfloor\sqrt{n}\rfloor$. In other words, $u(n) \leq \lfloor\sqrt{n}\rfloor$ (see Lemma 5), but $n - \lfloor\sqrt{n}\rfloor > \lfloor\sqrt{n}\rfloor$ for each $n > 2$.

The first for-loop in FareyLengthC computes the values of $|F_m|$ for $m \in M_1$ in $O(n^{3/4})$ time. However, this is not the fastest way of performing this computation because FareyLengthA (i.e., Algorithm 1) solves a similar problem in linear time with respect to the number of computed values of $|F_m|$. That is, instead of $O(n^{3/4})$ time it is possible to perform this computation in $O(\sqrt{n})$ time.

Simply speeding up the first for-loop in Algorithm 5 won't affect its overall time complexity class because the second for-loop still runs in $O(n^{3/4})$ time. The second for-loop uses the recursive formula for computing the values of $|F_m|$ for each m in the set M_2. Because M_2 is sparse, switching to a sieve-based algorithm instead of the recursive formula won't speed up the computation of the required values of $|F_m|$ for $m > \lfloor\sqrt{n}\rfloor$.

Nevertheless, it is possible to change the split between the sets M_1 and M_2 so that the sieve-based approach processes more values of m, including some values that are greater than $\lfloor\sqrt{n}\rfloor$. Because that approach computes the values of $|F_m|$ for each integer m in the designated interval, it would still compute $|F_m|$ for each $m \in M_1$. In other words, the modified algorithm can run faster despite computing more values of $|F_m|$ than required for computing $|F_n|$ using the recursive formula.

Let $M_1^*(x)$ be a function that maps a real number x to the following set:

$$M_1^*(x) = \{1, 2, \ldots, \lfloor x \rfloor\}. \tag{120}$$

Also, let $\hat{u}(x)$ be the following function:

$$\hat{u}(x) = \left\lfloor \frac{n}{\lfloor x \rfloor + 1} \right\rfloor. \tag{121}$$

Furthermore, let $M_2^*(x)$ be another function that maps a real number to a set, which is defined as follows:

$$M_2^*(x) = \left\{ \left\lfloor \frac{n}{\hat{u}(x)} \right\rfloor, \left\lfloor \frac{n}{\hat{u}(x)-1} \right\rfloor, \ldots, \left\lfloor \frac{n}{1} \right\rfloor \right\}. \tag{122}$$

Then, $M_1 = M_1^*(\sqrt{n})$ and $M_2 = M_2^*(\sqrt{n})$.

Using FareyLengthA we can compute $|F_m|$ for each $m \in M_1^*(x)$ in $O(x)$ time. Using FareyLengthC, we can compute $|F_m|$ for each $m \in M_2^*(x)$ in $O(g(x))$ time, where $g(x)$ is the following function:

$$g(x) = \sum_{m \in M_2^*(x)} \lfloor\sqrt{m}\rfloor = \sum_{j=1}^{\hat{u}(x)} \left\lfloor \sqrt{n/j} \right\rfloor. \tag{123}$$

The value of $g(x)$ can be bounded from above as follows:

$$g(x) = \sum_{j=1}^{\hat{u}(x)} \left\lfloor \sqrt{\frac{n}{j}} \right\rfloor \leq \sum_{j=1}^{\lfloor \frac{n}{x} \rfloor} \left\lfloor \sqrt{\frac{n}{j}} \right\rfloor \leq \sum_{j=1}^{\lfloor \frac{n}{x} \rfloor} \sqrt{\frac{n}{j}} = \sqrt{n} \sum_{j=1}^{\lfloor \frac{n}{x} \rfloor} \sqrt{\frac{1}{j}} = \sqrt{n} \sum_{j=1}^{\lfloor \frac{n}{x} \rfloor} \frac{1}{\sqrt{j}}. \tag{124}$$

Lemma 11 implies that the sum in the right-hand side of this inequality is in $O(\sqrt{\lfloor n/x \rfloor})$. In other words,

$$g(x) \leq \sqrt{n} \sum_{j=1}^{\lfloor \frac{n}{x} \rfloor} \frac{1}{\sqrt{j}} = \sqrt{n}\, O\big(\sqrt{\lfloor n/x \rfloor}\big) = \sqrt{n}\, O\big(\sqrt{n/x}\big) = O\big(n/\sqrt{x}\big). \tag{125}$$

The time complexity of computing $|F_m|$ for each $m \in M_1^*(x) \cup M_2^*(x)$ by an algorithm that uses the sieving approach to process the set $M_1^*(x)$ and the recursive approach to process $M_2^*(x)$ is in $O(f(x))$, where $f(x)$ is the following function:

$$f(x) = x + \frac{n}{\sqrt{x}} = x + n\,x^{-\frac{1}{2}}. \tag{126}$$

What is the optimal value of x for which $f(x)$ is minimized? Differentiating $f(x)$ leads to the following formula for its first derivative:

$$f'(x) = 1 - \frac{1}{2} n\,x^{-\frac{3}{2}}. \tag{127}$$

The equation $f'(x) = 0$ has only one root at $x = ({}^{n}\!/_{2})^{2/3}$. Multiplying this solution by a constant factor that is independent of n does not affect the time complexity class of the overall computation because any constant factor is subsumed by the big-O notation. For simplicity, the optimal split x^* can be set to $\lfloor n^{2/3} \rfloor$.

FAREYLENGTHD is the result of the modifications to FAREYLENGTHC described above. It replaces the first for-loop with FAREYLENGTHA in order to process the set $M_1^*(\lfloor n^{2/3} \rfloor)$. It also modifies the second for-loop to start from $v = \left\lfloor {}^{n}\!/\big(\lfloor \sqrt[3]{n^2} \rfloor + 1\big) \right\rfloor$ instead of $u = \left\lfloor {}^{n}\!/\big(\lfloor \sqrt{n} \rfloor + 1\big) \right\rfloor$ in order to compute $|F_m|$ for each m in the set $M_2^*(\lfloor n^{2/3} \rfloor)$. The resulting algorithm runs in $O(n^{2/3})$ time because each of these two sub-parts requires $O(n^{2/3})$ time. The space complexity of this algorithm is determined by the sieve-based approach that processes the set $M_1^*(\lfloor n^{2/3} \rfloor)$. Because this step requires holding arrays of length $\lfloor n^{2/3} \rfloor$ in memory, FAREYLENGTHD uses $O(n^{2/3})$ memory.

S13. Alternative Version of FareyLengthD

Algorithm S13 gives the pseudo-code for an alternative version of Algorithm 7 (i.e., FAREYLENGTHD). This version has the same run-time complexity and the same memory complexity as the one described in the main paper. It is shorter than the other algorithm, but updates more entries of the lookup table than necessary to compute $|F_n|$. It also obscures the link to Algorithm 8 (i.e., FAREYLENGTHE), which improves the space complexity. Additional details are provided in the main paper.

Algorithm S13. Alternative version of Algorithm 7. Runs in $O(n^{2/3})$ time and uses $O(n^{2/3})$ memory.

function FAREYLENGTHD2(n)
  $c \leftarrow$ ICBRT(n^2);
  $v \leftarrow \left\lfloor \frac{n}{c+1} \right\rfloor$;
  $F \leftarrow$ LOOKUPTABLE(n, $c+1$);
  $(P, L_p) \leftarrow$ LINEARSIEVE(c);
  $\varphi \leftarrow$ COMPUTETOTIENTS(c, L_p);
  $s \leftarrow 1$;
  for $m \leftarrow 1$ **to** c **do**
    $s \leftarrow s + \varphi[m]$;
    $F[m] \leftarrow s$;
  end for
  for $j \leftarrow v$ **down to** 1 **do**
    UPDATELOOKUPTABLE(F, $\lfloor n/j \rfloor$);
  end for
  return $F[n]$;
end function

S14. Computational Complexity Analysis of FareyLengthE

The following theorem proves the time and space complexity of FareyLengthE (i.e., Algorithm 8).

Theorem 21. *Algorithm 8 runs in $O(n^{2/3})$ and uses $O(\sqrt{n})$ memory.*

Proof. The time complexity of Algorithm 8 is in the same complexity class as Algorithm 7, i.e., $O(n^{2/3})$. More specifically, FareyLengthE starts by processing the integers in the interval $[1, \lfloor\sqrt{n}\rfloor]$ using the linear sieve and Algorithm S3. Next, the algorithm enumerates all $\lfloor\sqrt{n}\rfloor$-smooth numbers in the interval $[\lfloor\sqrt{n}\rfloor + 1, \lfloor n^{2/3}\rfloor]$ using Algorithm 3 and adds their totients to the corresponding elements of the array B. This step requires $O(n^{2/3})$ time and uses $O(\sqrt{n})$ memory. Next, the algorithm enumerates all numbers that are not $\lfloor\sqrt{n}\rfloor$-smooth in the interval $[\lfloor\sqrt{n}\rfloor + 1, \lfloor n^{2/3}\rfloor]$ using Algorithm 4 and also adds their totients to the corresponding elements of B. This step requires $O(n^{2/3})$ time and $O(\sqrt{n})$ memory because it uses the sieve of Atkin[19] to find the prime numbers between $\lfloor\sqrt{n}\rfloor+1$ and $\lfloor n^{2/3}\rfloor$. Finally, the algorithm computes the value of $|F_m|$ for each m in the set $\{\lfloor\frac{n}{v(n)}\rfloor, \lfloor\frac{n}{v(n)-1}\rfloor, \dots, \lfloor\frac{n}{1}\rfloor\}$ using the helper function. Similarly to FareyLengthD, this step requires $O(n^{2/3})$ time and $O(\sqrt{n})$ memory. Therefore, FareyLengthE runs in $O(n^{2/3})$ time and its space complexity is $O(\sqrt{n})$. ☐

The following three theorems prove that the computation in Algorithm 8 is correct. They show that the set of integers in the interval $[\alpha, \beta]$ that the algorithm enumerates is the correct set, where $\alpha = \lfloor\sqrt{n}\rfloor + 1$ and $\beta = \lfloor n/(v(n)+1)\rfloor$. The theorems also show that the formula for mapping k to the index i of the array element $B[i]$ is also correct for each integer k in $[\alpha, \beta]$.

The first theorem shows that the set $S(u(n), n)$, which consists of all integers j such that $\lfloor\frac{n}{j}\rfloor = u(n)$, includes the value of $\lfloor\sqrt{n}\rfloor + 1$. This implies that $\alpha = \lfloor\sqrt{n}\rfloor + 1$ is a suitable split point for switching between the linear sieve approach and enumeration of smooth and non-smooth integers. For this value of α the algorithm computes the value of $B[0]$ correctly because the split point is an element of $S(u(n), n)$ so that all integers in $S(u(n), n)$ are accounted for either by the computation of $|F_1|, |F_2|, \dots, |F_{\lfloor\sqrt{n}\rfloor}|$ using the linear sieving approach or by enumerating smooth and non-smooth numbers.

Theorem 22. *Let $S(k, n)$ be a set of all positive integers m such that the value of $\lfloor\frac{n}{m}\rfloor$ is equal to k. That is,*

$$S(k, n) = \{m \in \mathbb{N} \text{ s.t. } \lfloor\tfrac{n}{m}\rfloor = k\}. \tag{128}$$

Then, $\lfloor\sqrt{n}\rfloor + 1 \in S(u(n), n)$. Moreover, the set $S(u(n), n)$ is a contiguous range of integers such that

$$S(u(n), n) = \{\lfloor\tfrac{n}{u(n)+1}\rfloor + 1, \lfloor\tfrac{n}{u(n)+1}\rfloor + 2, \dots, \lfloor\tfrac{n}{u(n)}\rfloor\}, \tag{129}$$

$$\min S(u(n), n) = \lfloor\tfrac{n}{u(n)+1}\rfloor + 1 \le \lfloor\sqrt{n}\rfloor + 1 \le \max S(u(n), n) = \lfloor\tfrac{n}{u(n)}\rfloor. \tag{130}$$

Proof. Formula (129) follows from Lemma 4 after plugging $u(n)$ as the value of k in formula (29). Formula (130) follows from (129) and the definitions for the set $S(u(n), n)$ and the function $u(n)$. That is, $\lfloor\sqrt{n}+1\rfloor \in S(u(n), n)$ because $S(u(n), n)$ is a contiguous range of integers. Thus, $\lfloor\sqrt{n}+1\rfloor$ lies between its minimum and maximum elements. ☐

The second theorem shows that the endpoint $\beta = \lfloor\frac{n}{v(n)+1}\rfloor$ that Algorithm 8 uses as the upper limit for enumerating smooth and non-smooth numbers is the correct endpoint. That is, it shows that the enumeration process covers all elements of the sets $S(u(n)-1, n), S(u(n)-2, n), \dots, S(v(n)+1, n)$, which is required for computing the elements of the array B correctly.

Theorem 23. *The union of the sets $S(u(n)-1, n), S(u(n)-2, n), \dots, S(v(n)+1, n)$ is equal to the contiguous range of integers between $\lfloor\frac{n}{u(n)}\rfloor + 1$ and $\lfloor\frac{n}{v(n)+1}\rfloor$, where $v(n) = \lfloor n/(\lfloor\sqrt[3]{n^2}\rfloor+1)\rfloor$. More formally,*

$$\bigcup_{k=v(n)+1}^{u(n)-1} S(k, n) = \{\lfloor\tfrac{n}{u(n)}\rfloor + 1, \lfloor\tfrac{n}{u(n)}\rfloor + 2, \dots, \lfloor\tfrac{n}{v(n)+1}\rfloor\}. \tag{131}$$

Proof. This theorem follows from equation (29) in the statement of Lemma 4, which implies that the set $S(u(n)-1, n)$ is a contiguous range of integers that starts from $\lfloor\frac{n}{u(n)}\rfloor + 1$, the set $S(u(n)-2, n)$ is a contiguous range of integers that follows $S(u(n)-1, n)$ without any gaps in-between, and so forth until $S(v(n)+1, n)$. The lemma also shows that the maximum element of $S(v(n)+1, n)$ is equal to $\lfloor\frac{n}{v(n)+1}\rfloor$. ☐

The last theorem proves that the formula $i = u(n) - \lfloor\frac{n}{m}\rfloor$ that Algorithm 8 uses to compute the value of the index for the array B in the visitor function correctly identifies the zero-based indices of these elements. This theorem implies that $i = 0$ corresponds to $S(u(n), n)$, $i = 1$ corresponds to $S(u(n)-1, n)$, etc., until $i = w(n)$, which maps to $S(v(n)+1, n)$, where $w(n) = u(n) - v(n) - 1$.

Theorem 24. *Let m and n be two positive integers and let m be an element of the set $S(k, n)$. Then, $k = u(n) - i$, where $i = u(n) - \lfloor\frac{n}{m}\rfloor$.*

Proof. The proof follows from the definition of the set $S(k, n)$ in Lemma 4. That is, $m \in S(k, n)$ implies that $\lfloor\frac{n}{m}\rfloor = k$. Therefore, $i = u(n) - \lfloor\frac{n}{m}\rfloor = u(n) - k$. Thus, $k = u(n) - i$. ☐

S15. Results for the Number of CPU Instructions

In addition to the run time and memory usage results described in the main paper, we also measured the number of CPU instructions that the code executed (see Methods). Figure S4a visualizes these additional results for the C implementations of algorithms C, D, and E. Figure S4b shows the results obtained with the Python versions of these three algorithms. In both plots the value of n was varied from 10^0 to 10^{14} in increments of 0.5 on the decimal logarithm scale. Figure S4c lists the slopes and intercepts for six lines that were fitted to the six curves in (a) and (b) using least squares. The fitting procedure used the region between $n = 10^{10}$ and $n = 10^{14}$.

These results show that the number of CPU instructions agrees with the theoretical time complexities. Each slope is within 0.01 of the corresponding power of n in the time complexity class, i.e., $3/4$ for algorithm C and $2/3$ for algorithms D and E. More specifically, the slopes for the two programming language implementations of algorithm C are equal to 0.7494 and 0.7520, respectively. For algorithm D they are equal to 0.6672 and 0.6754. For algorithm E they are equal to 0.6655 and 0.6744.

This precise agreement with the theory suggests that the run time results reported in Figure 8 may be slightly higher than the theoretical predictions due to the practical aspects of running the code on modern processors. One of them could be increasingly inaccurate branch prediction as n increases. Another could be a greater number of cache misses for larger n. Because the time spent waiting for the cache to be updated does not affect the CPU instruction counter for the current process, this metric agrees with the theory very well.

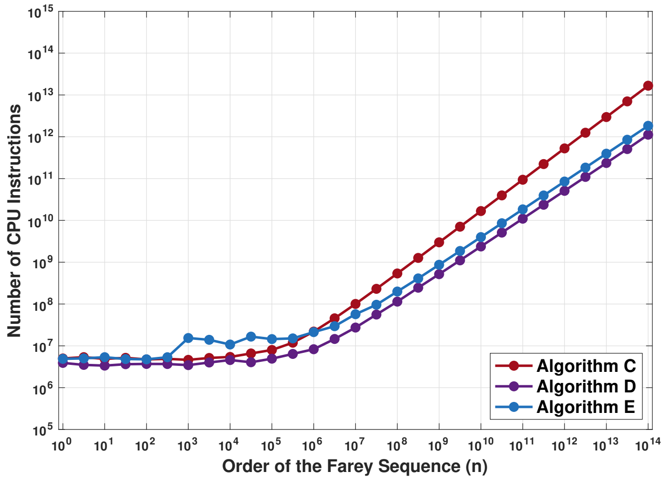

(a) Number of instructions executed by the C code.

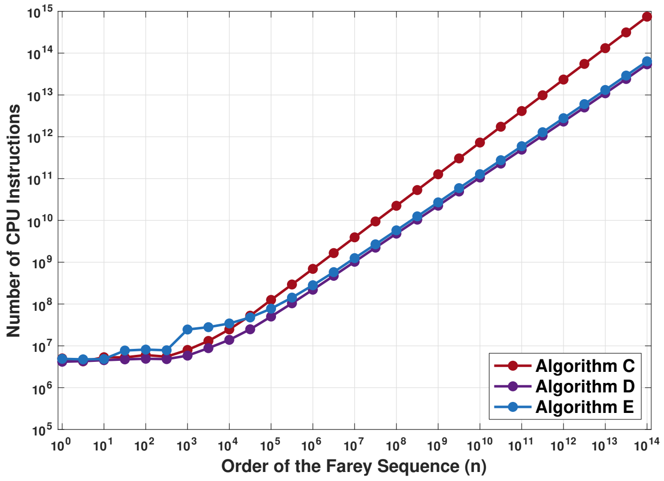

(b) Number of instructions executed by the Python code.

Alg.	C Code		Python Code	
	Slope	Intercept	Slope	Intercept
C	0.7494	2.7289	0.7520	4.3427
D	0.6672	2.7017	0.6754	4.2639
E	0.6655	2.9445	0.6744	4.3561

(c) Lines fitted to the curves in (a) and (b) for $n \geq 10^{10}$.

Figure S4: Number of CPU instructions executed by algorithms C, D, and E. The plots in (a) show the results for the C implementations of the algorithms. The results for the Python versions are shown in (b). The table in (c) lists the slopes and intercepts for lines fitted to the six curves in (a) and (b) in the region between $n = 10^{10}$ and $n = 10^{14}$.
